# RIPK1 is a negative mediator in Aquaporin 1-driven triple-negative breast carcinoma progression and metastasis

**DOI:** 10.1038/s41523-021-00261-5

**Published:** 2021-05-12

**Authors:** Zhuming Yin, Wenlin Chen, Jian Yin, Jingyan Sun, Qianrong Xie, Min Wu, Fanxin Zeng, Huiwen Ren

**Affiliations:** 1 Department of Breast Oncoplastic Surgery, Tianjin Medical University Cancer Institute and Hospital; National Clinical Research Center for Cancer; Key Laboratory of Breast Cancer Prevention and Therapy, Tianjin Medical University, Ministry of Education; Key Laboratory of Cancer Prevention and Therapy, Tianjin; Tianjin’s Clinical Research Center for Cancer; Sino-Russian Joint Research Center for Oncoplastic Breast Surgery, Tianjin, China; 2 Department of Breast Surgery, The First Affiliated Hospital of Xiamen University, Xiamen, China; 3 Department of Clinical Research Center, Dazhou Central Hospital, Dazhou, China; 4 Huaxi MR Research Center (HMRRC), Department of Radiology, West China Hospital of Sichuan University, Chengdu, China; 5 Department of Pharmacology, School of Basic Medical Sciences, Tianjin Medical University, Tianjin, China

**Keywords:** Breast cancer, Breast cancer, Oncogenes

## Abstract

The triple-negative breast carcinoma (TNBC) is the most aggressive subtype of breast cancer. In TNBC, Aquaporin 1 (AQP1), a water-transporting transmembrane protein, is aberrantly enriched in cytoplasm and causes tumor cell death evasion. However, the carcinogenetic bioactivities of cytoplasmic AQP1 cannot be attributed to the canonical “osmotic engine model”. In the present study, the receptor-interacting protein kinase 1 (RIPK1), a cell death regulator, was identified to negatively mediate AQP1-driven TNBC progression and metastasis. AQP1 overabundance and RIPK1 depletion occurred in TNBC, which were correlated with aggressive oncological features and poor prognosis. AQP1 bound with RIPK1, resulting in the inhibition of RIPK1/RIPK3/MLKL-mediated necroptosis and RIPK1/caspase-8/caspase-3-mediated apoptosis. Genetic inhibition of RIPK1 significantly exacerbated the pro-tumor effect of AQP1, while ectopic expression of RIPK1 notably blunted AQP1 signaling. Mechanistically, AQP1 binds to the D324 site of RIPK1, and facilitates RIPK1 cleavage and inactivation by excessively activating the caspase-8/RIPK1 negative feedback loop. RIPK1^D324K^ overexpression significantly prevented RIPK1 cleavage and weakened the aggressiveness of AQP1-enriched TNBC cells. Overall, our findings clarify the underlying mechanism of cytoplasmic AQP1-driven TNBC progression and metastasis, in which RIPK1 exerts an essential role as a negative mediator and exhibits the potential as a therapeutic target for TNBC.

## Introduction

The triple-negative breast carcinoma (TNBC) is the most aggressive subtype of breast cancer, which is characterized by lack expression of estrogen receptor (ER) and progesterone receptor (PR), and non-amplification of human epidermal growth factor receptor 2 (HER2)^1,2^. The triple-negative phenotype is universally acknowledged to be responsible for the higher rates of relapse, metastasis, and mortality compared to other breast cancer subtypes. However, systemic treatment against TNBC remains limited with the conventional chemotherapy as the mainstay, because patients with triple-negative disease do not benefit from endocrine therapy or anti-HER2-targeted therapy, which have been widely applied in the setting of ER/PR-positive or HER2-positive breast cancers with improved prognosis^1–3^. Even worse is that some TNBC subsets fail to respond to chemotherapy^4^, necessitating an in-depth understanding of TNBC-specific signaling pathways and a de novo exploration of biomarker-sensitive therapeutic strategies.

Previous studies have identified Aquaporin 1 (AQP1), a water-transporting transmembrane protein, to be upregulated in TNBC and associated with the tumor development and progression^5–7^. AQP1 may behave as an oncogenic biomarker for numerous types of cancer that is able to sustain tumor pathogenesis through facilitating cell proliferation, migration, and angiogenesis^8–11^. Possible mechanisms may include the resistance to cell death that predisposes AQP1-expressing cells toward growth and metastasis^12–14^. Unfortunately, the exact effect of AQP1 on TNBC cell death evasion and the specific signaling pathway are still far from understood.

The receptor-interacting protein kinase 1 (RIPK1) is a central regulatory molecule in cell fate decision with dual bioactive properties as a coalescence of caspase-8-mediated apoptosis and a guardian of RIPK3-mediated necroptosis^15–21^. Thus, the RIPK1 kinase has emerged as a promising therapeutic target for a wide spectrum of neurological, cardiovascular, renal, and hepatic disorders, and infectious and inflammatory diseases^22,23^. In addition, compelling evidence has documented that RIPK1 may be a bona fide biomarker and a novel target for cancer diagnosis and therapy by manipulating cellular demise^24–27^. However, the role of RIPK1 in breast cancer oncogenesis and development remains one of the major unsolved issues, which may lead to a better understanding of AQP1-related TNBC cell death escape.

In the current study, we report the aberrant expression of AQP1 and RIPK1 in TNBC that are associated with different prognoses. Further, we validate the interaction of AQP1 and RIPK1, and the suppressive effect of RIPK1 on AQP1-driven TNBC progression and metastasis. Finally, we identify the underlying mechanism of TNBC cell death resistance that AQP1 binds to the D324 site of RIPK1, and facilitates RIPK1 cleavage by promoting the caspase-8/RIPK1 negative feedback loop. Our results provide new insights into the essential role of RIPK1 as a negative mediator in AQP1-driven TNBC progression and metastasis, which might represent a potential therapeutic target for TNBC.

## Results

### AQP1 is upregulated in triple-negative breast carcinoma and associated with poor survival

To determine the expression profile of AQP1 in invasive ductal breast carcinoma (IDC), the Finak’s cohort^28^ was analyzed using the Oncomine database. Compared with the normal breast parenchyma, over ninefold upregulation of AQP1 in IDC was revealed with statistical significance (Supplementary Fig. 1A, *p* < 0.001). We next explored the subtype-specific abundance of AQP1 by deciphering the the Cancer Genome Atlas (TCGA), Genotype-Tissue Expression (GTEx) datasets (GSE1456/GSE6532/GSE7390). The result identified significantly higher expression level of AQP1 in TNBC than that in other subtypes (Supplementary Fig. 1B, *p* < 0.01).

**Fig. 1:**
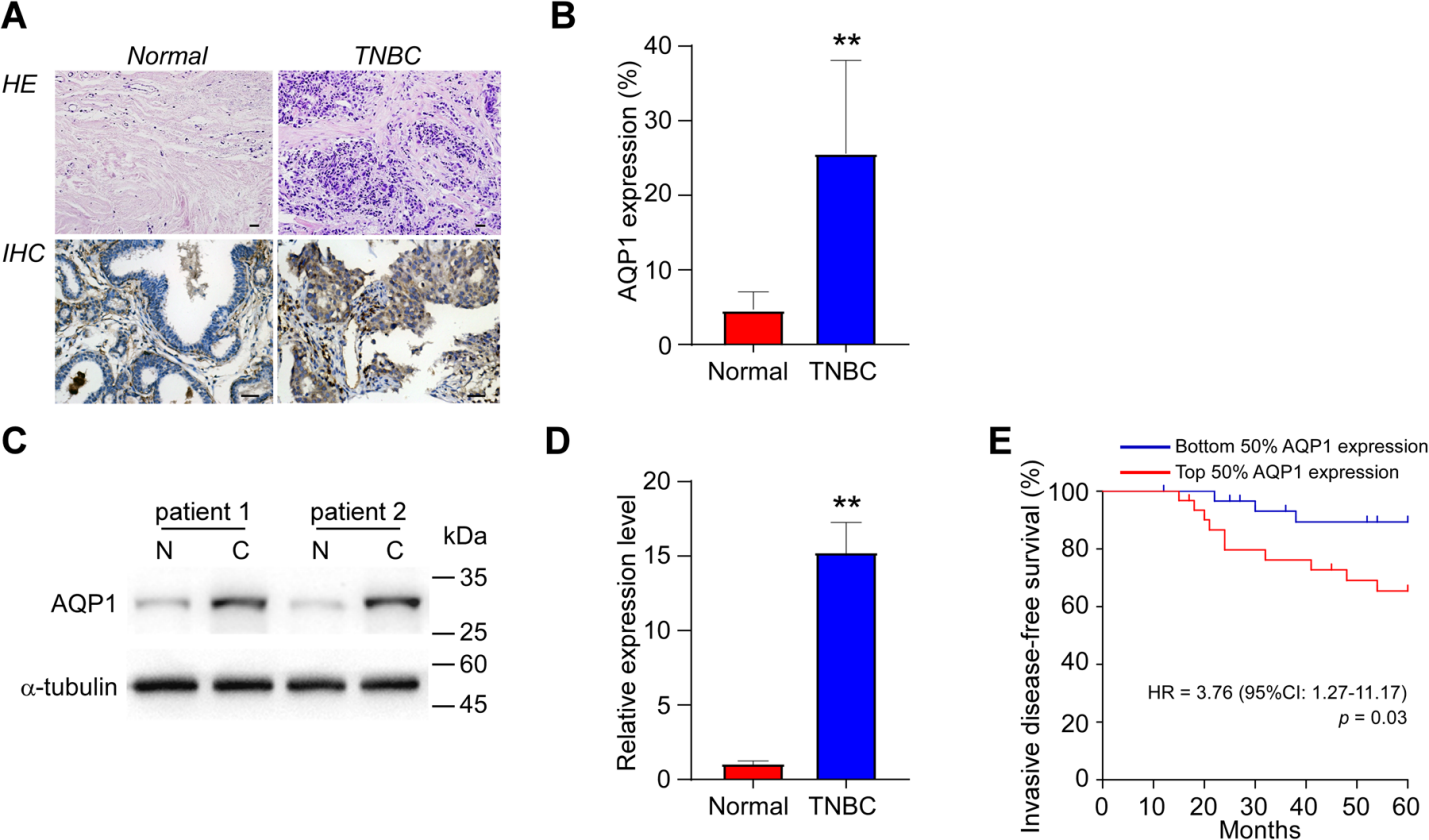
AQP1 is upregulated in TNBC and associated with poor survival. **A** Representative images of hematoxylin–eosin (*above*, HE, ×100 magnification) and anti-AQP1 immunohistochemistry (*below*, IHC, ×200 magnification) staining of TNBC and normal breast tissue. *Scale bar*: 40 μm. **B** Quantification of the AQP1-positive cell percentage in TNBC and normal breast tissue (*n* = 62). **C** Representative blotting of Western blot analysis of TNBC and normal breast tissue. *Abbreviations*: N, normal breast tissue; C, TNBC. **D** Quantification of relative expression levels of AQP1 in TNBC and normal breast tissue (*n* = 62). **E** Kaplan–Meier survival plot showing the invasive-disease-free survival of TNBC patients in the current cohort stratified by dichotomic expression levels of AQP1 (*n* = 31 for each group). *Note*: **p < 0.01.

To further confirm the subtype-specific expression pattern in big data analyses, tumor samples, and para-carcinoma normal tissues were harvested from 62 patients, who were pathologically diagnosed as unilateral TNBC at two tertiary medical centers. Consistent with our expectation, AQP1 abundance in TNBC was remarkably higher than normal control in both immunohistochemistry staining and Western blot analysis (Fig. 1A–D, *p* < 0.01). Intriguingly, AQP1 was observed with dominant cytoplasmic expression in TNBC cells instead of its common location on the cell membrane in normal breast parenchyma (Fig. 1A), indicating that the cytoplasmic AQP1 is one of the main culprits in TNBC tumorigenesis. Moreover, the clinical cases were divided into high-expression (*n* = 31) and low-expression (*n* = 31) groups, according to the immunostaining score. As shown in Table 1, the age, BMI, tumor size, histological grade, and surgical choice did not differ in parallel with AQP1 expression profile (*p* > 0.05); while the lymph node metastasis and radiotherapy occurred more frequently in the high-expression group (*p* < 0.01), suggesting that AQP1 abundance is strongly associated with TNBC progression. Meanwhile, to find out the association between AQP1 expression and the prognosis of TNBC, the invasive-disease-free survival rate was calculated in our cohort. The result demonstrated that TNBC patients with high tumor expression had less favorable survival compared with patients, whose tumors expressed a low level of AQP1 (Fig. 1E; hazard ratio, 3.76; *p* = 0.03). Taken together, the upregulation of AQP1 plays an important role in promoting TNBC tumorigenesis and progression, and represents poor prognosis.

**Table 1:** Frequency and distribution of AQP1 expression in association with established sociodemographic and oncological features of TNBC patients.

	Overall (*n* = 62)^a^	AQP1 expression			RIPK1 expression		
		Low (*n* = 31)	High (*n* = 31)	*p* Value^b^	Low (*n* = 31)	High (*n* = 31)	*p* Value^b^
**Age**				0.437			0.796
≤50 years	37 (59.7)	20	17		18	19	
>50 years	25 (40.3)	11	14		13	12	
**BMI**				0.167			0.490
≤24 kg/m^2^	52 (83.9)	28	24		25	27	
>24 kg/m^2^	10 (16.1)	3	7		6	4	
**Tumor size**				0.118			0.479
≤2 cm	11 (17.7)	4	7		7	4	
2–5 cm	26 (41.9)	17	9		11	15	
>5 cm	25 (40.4)	10	15		13	12	
**Lymph node status**				0.005			0.022
Negative	29 (46.8)	20	9		10	19	
Positive	33 (53.2)	11	22		21	12	
**Histological grade**				0.445			0.203
1	0 (0)	0	0		0	0	
2	29 (46.8)	16	13		12	17	
3	33 (53.2)	15	18		19	14	
**Surgery**				0.374			0.038
Breast conserving	15 (24.2)	9	6		4	11	
Ablative	47 (75.8)	22	25		27	20	
**Radiotherapy**				0.002			0.013
No	19 (30.6)	15	4		5	14	
Yes	43 (69.4)	16	27		26	17	

^a^The data are displayed as No. (%).

^b^*χ*^2^ *test*.

*Abbreviations*: TNBC, triple-negative breast cancer; BMI, body mass index.

### RIPK1 is downregulated in triple-negative breast carcinoma and associated with enhanced survival

**Fig. 2:**
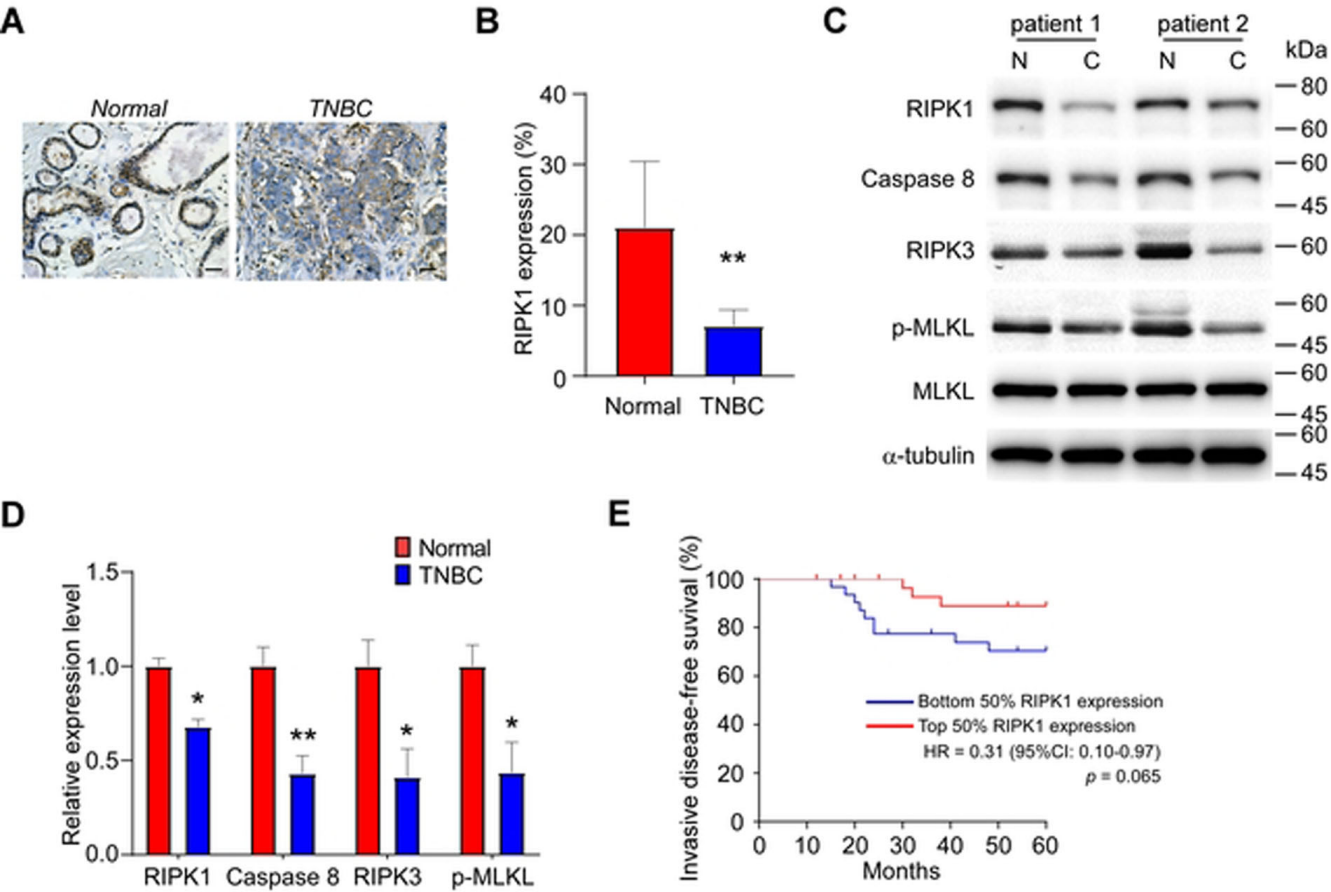
RIPK1 is downregulated in TNBC and associated with enhanced survival. **A** Representative images of anti-RIPK1 immunohistochemistry staining of TNBC and normal breast tissue (×200 magnification). *Scale bar*: 40 μm. **B** Quantification of the RIPK1-positive cell percentage in TNBC and normal breast tissue (*n* = 62). **C** Representative blotting of Western blot analysis of TNBC and normal breast tissue. *Abbreviations*: N, normal breast tissue; C, TNBC. **D** Quantification of relative expression levels of RIPK1, Caspase-8, RIPK3, and phospho-MLKL (p-MLKL) in TNBC and normal breast tissue (*n* = 62). **E** Kaplan–Meier survival plot showing the invasive-disease-free survival of TNBC patients in the current cohort stratified by dichotomic expression levels of AQP1 (*n* = 31 for each group). *Note*: *p < 0.05, **p < 0.01.

To investigate the RIPK1 expression in IDC, the Curtis’s cohort^29^ was retrieved from the Oncomine database. Contrary to our expectation, a modest (1.098-fold) but significant increase in RIPK1 expression intensity in IDC was observed in contrast to the normal breast tissue (Supplementary Fig. 2A, *p* < 0.001). However, we found RIPK1 was strikingly downregulated in TNBC compared with other breast carcinoma subtypes based on the analysis of the TCGA and GTEx datasets (Supplementary Fig. 2B, *p* < 0.01). Furthermore, a notable TNBC-specific reduction of RIPK1 abundance in our cohort was identified by immunohistochemistry staining and Western blot analysis (Fig. 2A–D, *p* < 0.05). Meanwhile, the relative expression levels of caspase-8, RIPK3, and phospho-MLKL, known as critical execution molecules of programmed cell death, decreased drastically compared with normal breast tissues (Fig. 2C, D, *p* < 0.05), indicating that the compromised RIPK1-mediated programmed cell death is associated with TNBC tumorigenesis.

Next, we examined the frequency of different RIPK1 expression levels in the current TNBC cohort. There was no statistically significant change pertaining to age, BMI, tumor size, and histological grade (Table 1, *p* > 0.05); while negative axillary lymph node or earlier clinical tumor staging was more likely to be distributed in the high-expression group (*p* < 0.05), following the treatment decision of breast conservation and non-radiotherapy (*p* < 0.05). The invasive-disease-free survival analysis demonstrated enhanced TNBC-specific survival rate in patients with higher tumor expression of RIPK1 (Fig. 2E; hazard ratio, 0.31; *p* = 0.065). Collectively, these findings imply that RIPK1 exerts an inhibitive role in TNBC development and a positive role in patient survival.

### AQP1 interacts with RIPK1 in triple-negative breast carcinoma

**Fig. 3:**
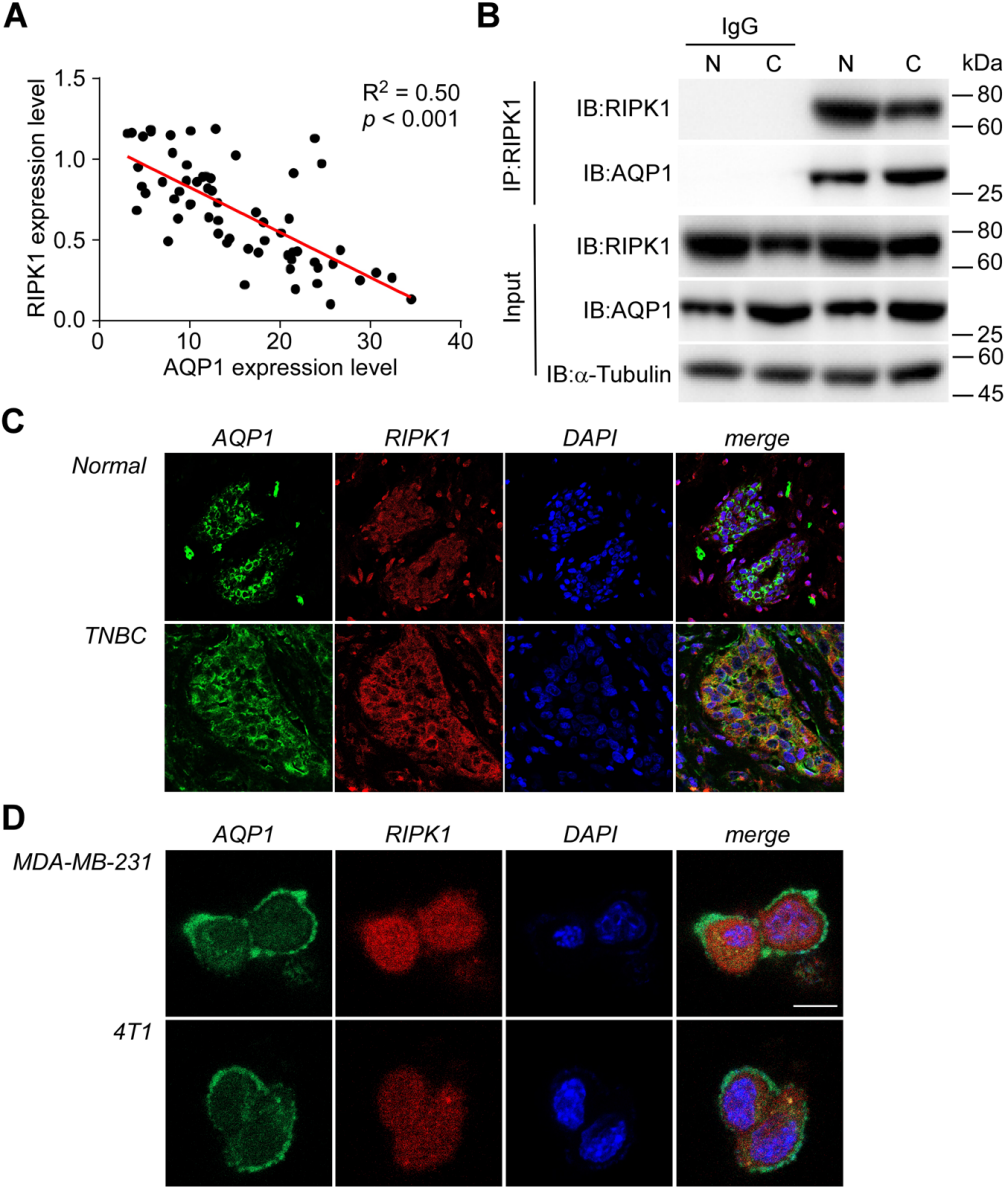
AQP1 binds with RIPK1 in TNBC. **A** Linear regression graph. Strong negative correlation (*R*^2^ = 0.50, *p* < 0.001) was revealed in TNBC between the abundance of AQP1 and RIPK1 detected by Western blotting. **B** Co-immunoprecipitation of RIPK1 from TNBC and normal breast tissue showing AQP1 binds with RIPK1. *Abbreviations*: N, normal breast tissue; C, TNBC. **C** Representative images of anti-AQP1 (green) and anti-RIPK1 (red) immunofluorescence staining of TNBC and normal breast tissue (×400 magnification). Orange/yellow fluorescence in the merged images represents the colocalization of AQP1 and RIPK1. **D** Representative images of anti-AQP1 (green) and anti-RIPK1 (red) immunofluorescence staining of MDA-MB-231 and 4T1 cells stably overexpressing AQP1 and RIPK1. Orange/yellow fluorescence in the merged images represents the colocalization of AQP1 and RIPK1. *Scale bar*: 20 μm.

As both AQP1 accumulation and RIPK1 depletion coexist in TNBC, we first looked for a correlation between the expression profiles of them in the tumor samples detected by Western blotting. The linear regression analysis showed a strong negative correlation between the abundance of AQP1 and RIPK1 (Fig. 3A, *R*^2^ = 0.50, *p* < 0.001). To clarify this correlation, the co-immunoprecipitation and mass spectrum assays were performed in the clinical specimens. The results supported the existence of interaction between AQP1 and RIPK1 in TNBC (Fig. 3B, Supplementary Fig. 3, and Supplementary Table 1). Moreover, the colocalization of AQP1 and RIPK1 was observed specifically in TNBC in contrast to normal breast tissues (Fig. 3C).

Because AQP1 is expressed in both membrane and cytoplasm of breast cancer cells^6,30^, but RIPK1 is exclusively localized in the cytoplasm, the localization of AQP1 and RIPK1 in MDA-MB-231, a human TNBC cell line, and 4T1, a mouse TNBC cell line, was assessed respectively by immunofluorescence staining. To avoid the distinction of endogenous expression, the cells were both AQP1- and RIPK1-overexpressed using a lentivirus infection system. Under an immunofluorescence confocal microscope, the cytoplasm was determined as the main field where the AQP1–RIPK1 complex assembles in both cell lines (Fig. 3D).

### Inhibition of RIPK1 is required for AQP1-driven TNBC cell proliferation, migration, and invasion in vitro

**Fig. 4:**
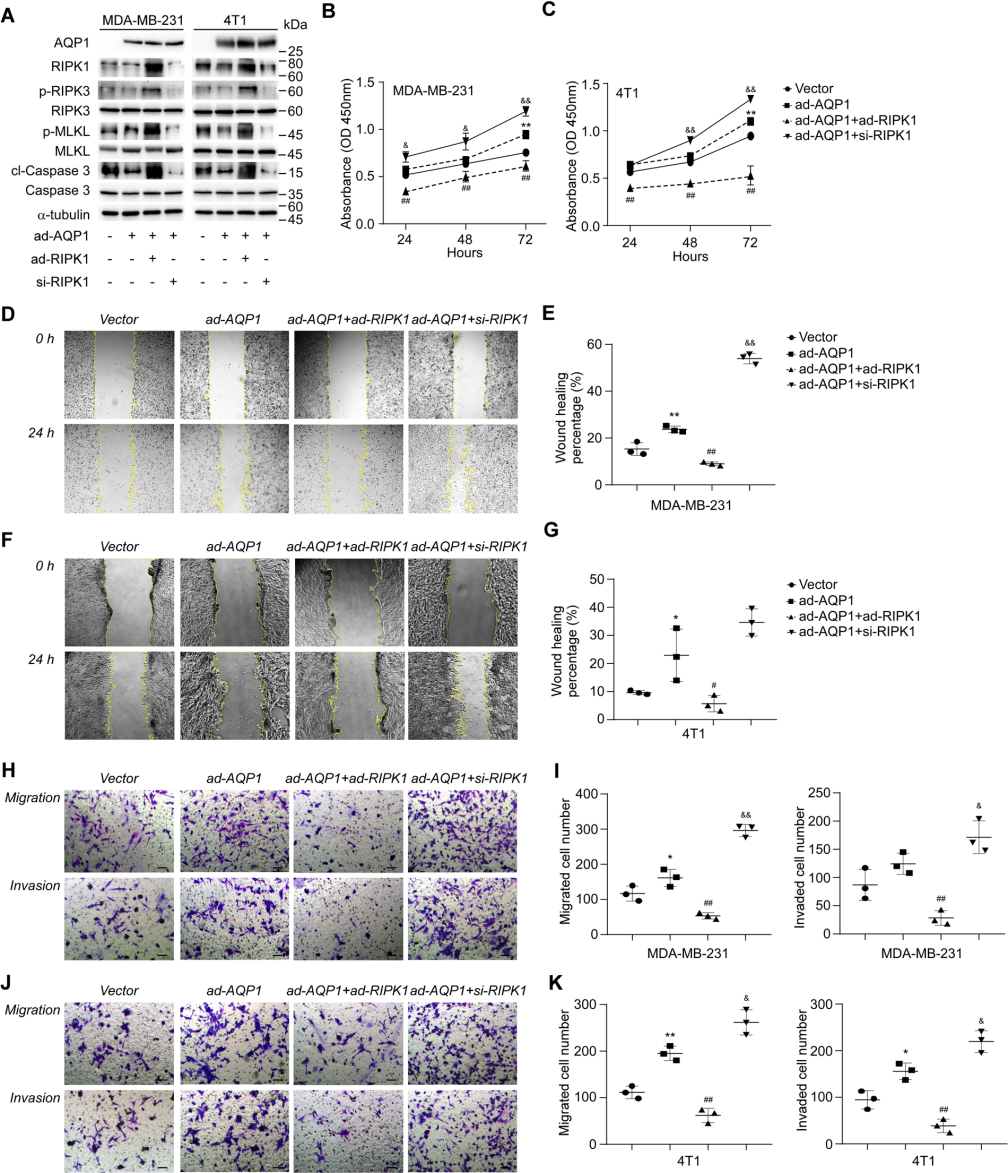
Inhibition of RIPK1 is required for AQP1-driven TNBC cell proliferation, migration, and invasion in vitro. **A** Representative blotting of Western blot analysis of MDA-MB-231 and 4T1 cells transfected with empty vector, AQP1-3×FLAG, RIPK1-HA, and/or RIPK1-siRNA. **B** Relative viability of indicated MDA-MB-231 cells at 24, 48, and 72 hours after cell seeding. The cell viability is displayed by the absorbance at 450 nm wavelength in the CCK-8 assay (*n* = 3). **C** Same experiment as in **B**, but using 4T1 cells (*n* = 3). **D** Representative images of indicated MDA-MB-231 cells in the wound healing assay at 0 and 24 hours after micropipette scratching (×100 magnification). **E** Quantification of the wound healing percentage at 24 hours after micropipette scratching in indicated MDA-MB-231 cell cultures (*n* = 3). **F**, **G** Same experiment as in **D** and **E**, but using 4T1 cells (*n* = 3). **H** Representative images of indicated MDA-MB-231 cells in the transwell migration and invasion assays (×200 magnification). *Scale bar*: 100 μm. **I** Quantification of migrated and invaded cells for indicated MDA-MB-231 cells (*n* = 3). **J**, **K** Same experiment as in **H** and **I**, but using 4T1 cells (*n* = 3). *Abbreviations*: ad-AQP1, AQP1 overexpression; ad-RIPK1, RIPK1 overexpression; si-RIPK1, RIPK1 knockdown with siRNA. *Note*: *p < 0.05 and **p < 0.01 vs. empty-vector control; ^#^*p* < 0.05 and ^##^*p* < 0.01 vs. ad-AQP1 group; ^&^*p* < 0.05 and ^&&^*p* < 0.01 vs. ad-AQP1 group.

To investigate the role of AQP1 in TNBC carcinogenesis and metastasis in vitro, the MDA-MB-231 and 4T1 cells stably overexpressing AQP1 were generated and verified by Western blot analysis (Fig. 4A). Both of the TNBC cells did not have endogenous expression of AQP1. The relative abundance of phospho-RIPK3, phospho-MLKL, and cleaved caspase-3, known as key mediators of necroptosis and apoptosis, was revealed to be downregulated in AQP1-overexpressing cells compared with that in the vector control, indicating that RIPK1-dependent programmed cell death was undermined by AQP1. The cell counting kit-8 (CCK-8) assay demonstrated a significant increase in the viability of both cell lines with AQP1 upregulation compared with the empty-vector group (Fig. 4B, C, *p* < 0.01 for both cell lines), suggesting a tight link between enhanced AQP1 expression and TNBC cell growth. Meanwhile, AQP1 overexpression promoted TNBC cell migration ability, as demonstrated with notable elevation of wound healing percentage (Fig. 4D–G, *p* < 0.01 for MDA-MB-231 and *p* < 0.05 for 4T1). The colony formation assays showed more migrated and invaded cells in AQP1-overexpressing groups than the cells in vector control group (Fig. 4H–K, *p* < 0.05 for MDA-MB-231 migration group and 4T1 invasion group, *p* < 0.01 for 4T1 migration group), indicating AQP1 raises the aggressiveness of TNBC cells, and may positively engage in TNBC progression and metastasis.

As increased AQP1 was accompanied by downregulation of RIPK1, it is hypothesized that RIPK1 dictates the oncogenic properties of AQP1 in TNBC. Thus the gain-of-function and loss-of-function approaches to overexpress and silence RIPK1 were employed to prove the assumption. As shown in Supplementary Fig. 4, both silencing RNA candidates resulted in over 90% knockdown of RIPK1 in MDA-MB-231 and 4T1 cells. The downregulation of phospho-RIPK3, phospho-MLKL, and cleaved caspase-3 in AQP1-expressing cells were restored by ectopic expression of RIPK1; while silencing RIPK1 further inactivated those downstream cell death mediators (Fig. 4A). In contrast to AQP1-expressing cells, the absorbance at 450 nm detected by CCK-8 assay was revealed with a drastic decrease in double-transgenic cells of AQP1 and RIPK1, and a significant increase in RIPK1-knockdown AQP1 transgenic cells at 24, 48, and 72 hours (Fig. 4B, C, *p* < 0.01 for gain-of-function cell lines, *p* < 0.05 for loss-of-function cell lines). On the other hand, RIPK1-overexpressing cells exhibited significantly lower wound healing percentage in the scratch assay (Fig. 4D–G, *p* < 0.01 for MDA-MB-231 and *p* < 0.05 for 4T1) and weaker migratory capacity in transwell insert-based cell migration assay with or without an extracellular matrix barrier (Fig. 4H–K, *p* < 0.01), though they also possessed additional AQP1 expression. By contrast, AQP1-driven TNBC cell migration and invasion were dramatically exacerbated in the absence of RIPK1, displayed by accelerated wound healing (Fig. 4D–G, *p* < 0.01 for MDA-MB-231) and improved cell penetration ability (Fig. 4H–K, *p* < 0.05 for MDA-MB-231 invasion group and both 4T1 groups, *p* < 0.01 for MDA-MB-231 migration group). To recapitulate, RIPK1 acts as a brake that arrests AQP1 signaling in TNBC cell proliferation, migration, and invasion in vitro.

### Upregulation of RIPK1 attenuates AQP1-driven TNBC progression and lung metastasis in vivo

**Fig. 5:**
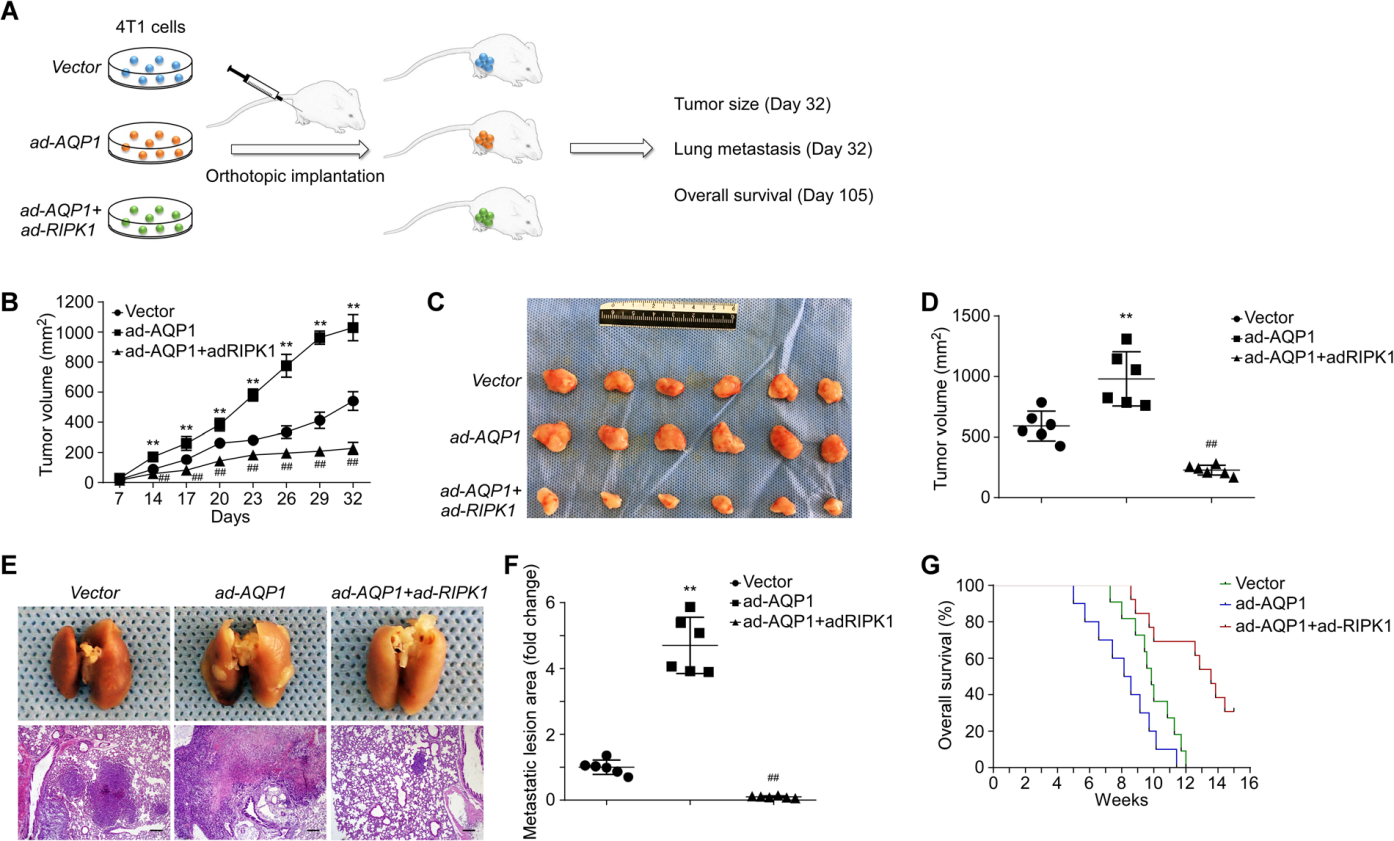
Upregulation of RIPK1 attenuates AQP1-driven TNBC progression and lung metastasis in vivo. **A** Flow chart of the in vivo orthotopic breast carcinoma model establishment and experiments. **B** Statistical analysis of consecutive tumor volume measurements until 32 days after 4T1 cell implantation (*n* = 6 for each group). **C** Representative images of tumors in empty-vector control, AQP1-overexpressing, AQP1–RIPK1-overexpressing 4T1 cell transplanted BALB/c mice. **D** Statistical analysis of the final tumor volume measurements at 32 days after 4T1 cell implantation (*n* = 6 for each group). **E** Representative images of gross lung and micrometastatic nodules (×40 magnification) from empty-vector control, AQP1-overexpressing, AQP1–RIPK1-overexpressing 4T1 cell transplanted BALB/c mice. *Scale bar*: 200 μm. **F** Statistical analysis of the metastatic lesion area measurements at 32 days after 4T1 cell implantation (*n* = 6 for each group). **G** Kaplan–Meier survival plot showing the overall survival of BALB/c mice orthotopically injected with empty-vector control, AQP1-overexpressing, AQP1–RIPK1-overexpressing 4T1 cells (*n* = 11 for each group). *Abbreviations*: ad-AQP1, AQP1 overexpression; ad-RIPK1, RIPK1 overexpression. *Note*: **p < 0.01 vs. empty-vector control; ^##^*p* < 0.01 vs. ad-AQP1 group.

To further validate the inhibitive effect of RIPK1 on AQP1-driven TNBC development, an orthotopic breast carcinoma mouse model was established by injecting transgenic 4T1 cells into the mammary fat pad of BALB/c mice (Fig. 5A). The volume of AQP1 transgenic tumors started to grow significantly larger (Fig. 5B, *p* < 0.01) at 14 days after transplantation than the vector control, and the trend lasted until the end of observation on the 32^nd^ day. On the contrary, RIPK1 overexpression remarkably reduced the size of tumors to an extent even lower than the vector control from day 14 to day 32, though they were also AQP1 enriched (Fig. 5B, *p* < 0.01). In addition, the final measurement of the tumors dissected from those mice identified a noted distinction among the three groups (Fig. 5C, D, *p* < 0.01), suggesting the carcinogenetic activity of AQP1 in TNBC can be kept in check by RIPK1, which is consistent with the in vitro findings.

Next, the effect of RIPK1 on AQP1-induced spontaneous lung metastasis of TNBC were examined. 4T1 cells with AQP1 overexpression showed significant enhancement of the migratory activity in vivo compared with the vector control, represented by increased lung metastatic lesions (Fig. 5E, F, *p* < 0.01). However, the lung metastases declined strikingly in the double-transgenic group compared to that in AQP1-expressing group (Fig. 5E, F, *p* < 0.01), demonstrating that the promotional effect of AQP1 on TNBC progression and metastasis in vivo was abolished by the upregulation of RIPK1. Moreover, 15-week consecutive observations revealed lower overall survival rate of mice injected with AQP1 transgenic cells than that of control mice (Fig. 5G; hazard ratio, 2.0; *p* = 0.08). By contrast, the mice bearing double-gene-overexpressing cells exhibited considerably longer survival time than the mice in AQP1 group (Fig. 5G; hazard ratio, 0.23; *p* < 0.001), indicating an improved prognosis of TNBC with high expression level of RIPK1. Overall, these findings proved that RIPK1 renders TNBC less susceptible to AQP1-triggered progression and lung metastasis in vivo.

### AQP1 binds to the D324 site of RIPK1 and excessively activates caspase-8-mediated RIPK1 cleavage

**Fig. 6:**
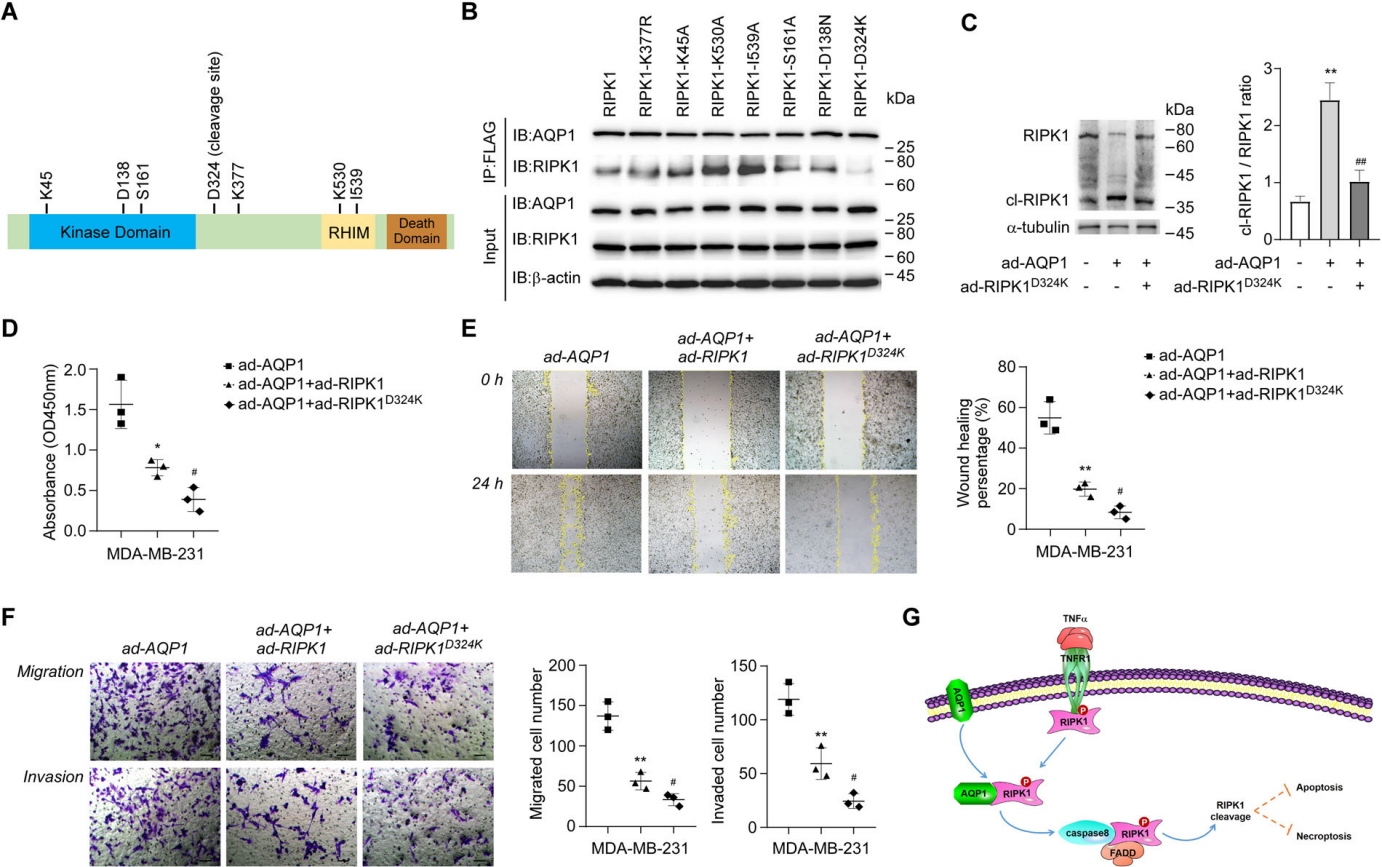
AQP1 binds to the D324 site of RIPK1 and excessively activates caspase-8-mediated RIPK1 cleavage. **A** Schematic diagram of RIPK1 structure and functional sites. **B** Co-immunoprecipitation of FLAG in HEK-293T cells expressing AQP1-3×FLAG transformed with plasmids expressing RIPK1, RIPK1-K377R, RIPK1-K45A, RIPK1-K530A, RIPK1-I539A, RIPK1-S161A, or RIPK1-D324K. RIPK1-D324K was revealed with reduced interaction with AQP1. **C** Representative blotting of Western blot analysis of RIPK1 and cleaved RIPK1 (cl-RIPK1) in MDA-MB-231 cells stably overexpressing AQP1 and RIPK1^D324K^. Statistical analysis of the cl-RIPK1/RIPK1 ratio showed significant difference among empty-vector control, AQP1-overexpressing, AQP1–RIPK1^D324K^-overexpressing cells (*n* = 3). **D** Relative viability of indicated MDA-MB-231 cells at 72 hours after cell seeding. The cell viability is displayed by the absorbance at 450 nm wavelength in the CCK-8 assay (*n* = 3). **E** Representative images and quantification of indicated MDA-MB-231 cells in the wound healing assay at 0 and 24 hours after micropipette scratching (×100 magnification, *n* = 3). **F** Representative images and quantification of indicated MDA-MB-231 cells in the transwell migration and invasion assays (×200 magnification, *n* = 3). *Scale bar*: 100 μm. **G** Schematic model of the role of cytoplasmic AQP1 in regulating TNBC cell apoptosis and necroptosis by facilitating caspase-8-mediated RIPK1 cleavage. *Abbreviations*: RHIM, receptor-interacting protein homotypic interacting motif; ad-AQP1, AQP1 overexpression; ad-RIPK1 RIPK1, overexpression; ad-RIPK1^D324K^, RIPK1^D324K^ overexpression. *Note:* **p < 0.01 vs. empty-vector control and ^##^*p* < 0.01 vs. ad-AQP1 group for **C**; *p < 0.05, **p < 0.01 vs. ad-AQP1 group; ^#^*p* < 0.05 vs. ad-AQP1 + ad-RIPK1 group for **D**–**F**.

Although our study has clarified the underlying mechanism of AQP1–RIPK1 complex in TNBC tumorigenesis and progression, it remains elusive how the complex affects downstream cell death signaling. To gain further insights into the interaction of AQP1 and RIPK1, seven-point mutation constructs of RIPK1 were generated, including plasmids targeting the kinase activity (K45A, D138N, and S161A), caspase-8-mediated cleavage (D324K), ubiquitination (K377R), and binding activity with RIPK3 (K530A and I539A). The mutants were immunoprecipated with AQP1-3×FLAG in HEK-293T cells. The results showed RIPK1 with D324K point mutant reduced the affinity of RIPK1 with AQP1 (Fig. 6A, B).

Because the previous literature demonstrated that D324 site-specific RIPK1 cleavage impinges on cell death and thus promotes cell survival^31–33^, the amount of cleaved RIPK1 (cl-RIPK1) was tested in MDA-MB-231 cells stably overexpressing AQP1 and RIPK1^D324K^. In contrast to the vector control, AQP1 transgenic cells presented significantly higher cl-RIPK1/RIPK1 ratio (Fig. 6C, *p* < 0.01). However, the activity of AQP1 in TNBC cell death evasion was blocked by the mutation of RIPK1 on D324 site, displayed by decreased cl-RIPK1/RIPK1 ratio, lowered absorbance at 450 nm in CCK-8 assay, reduced wound healing percentage in scratch assay, and weakened cell penetration capacity in transwell insert-based cell migration assay (Fig. 6C–F, *p* < 0.01). Moreover, compared with double-transgenic cells, RIPK1^D324K^ overexpression alone presented less cl-RIPK1/RIPK1 ratio, but identical capacity of cell survival, migration, and invasion (Supplementary Fig. 5, *p* > 0.05), suggesting that AQP1-driven TNBC progression and metastasis can be completely inhibited by loss of D324 site of RIPK1. It is also reflected by the fact that the viability and migration and invasion ability of AQP1-enriched cells were further mitigated in RIPK1^D324K^ overexpression group, compared with that in wild-type RIPK1-transgenic group (Fig. 6D–F, *p* < 0.05). Altogether these findings highlight the molecular mechanism of AQP1 in TNBC cell death resistance by overactivation of caspase-8-mediated RIPK1 cleavage (Fig. 6G).

## Discussion

Although the death rates have declined by over 30% since 1990^34^, breast cancer remains life-threatening for women around the world, because the survival disparities persist among the intrinsic subtypes. The TNBC, characterized by absence of ER/PR/HER2, favors tumor progression and metastasis, thus resulting in more cancer-related deaths^1,2^. Nevertheless, no specific therapy apart from cytotoxic chemotherapy has been available to date for the systemic intervention of TNBC due to a lack of appropriate therapeutic targets and insufficient information on their mechanisms. In the present study, we demonstrate for the first time the interaction and mutual regulation of AQP1 and RIPK1, as a novel signaling pathway to better understand the mechanisms of TNBC progression and metastasis.

AQP1 is a member of selective channels for water transport with the well-established role in maintaining tissue water balance and osmotic gradients^11^. It has been identified as an oncogene associated with multiple neoplasms, including breast cancer^9,14,23,27,30^. The current study revealed that AQP1 is upregulated in TNBC and strongly correlated with poor prognosis, which is consistent with the results of previous pathological investigations^6,7^. Intriguingly, we found increased expression of AQP1 in cytoplasm, which runs in parallel with lymph node metastasis. This finding is in accordance with previous reports indicating the positive correlation of cytoplasmic intensity of AQP1 and high-risk oncological features of TNBC^6,30^. In the controlled laboratory setting, AQP1-expressing cells proliferate and migrate more extensively in contrast to control cells, leading to significantly larger tumors and more spontaneous lung metastases in vivo, suggesting that AQP1 plays a crucial role in the acquisition of the aggressive phenotype of TNBC.

The classical paradigm of AQP-induced cell growth and invasion has been proposed as AQP polarization, which facilitates the cell morphology change and directional migration by orchestrating AQP-dependent transmembrane water/ion flow in an osmotic gradient^5,8,35^. This “osmotic engine model”^36^ provides a perfect explanation on the mechanism of membranous AQP1-modulated tumor migration and angiogenesis. However, the function of cytoplasm-enriched AQP1 remains to be determined, which may likely be the key to deciphering the mechanisms of TNBC progression and metastasis. Recently, the resistance to programmed cell death, including apoptosis and necroptosis, has been identified by emerging evidence as a putative mechanism of cytoplasmic AQP1-driven tumor proliferation and cancer spread^12–14^. In the present study, we made further progress by demonstrating that RIPK1, a cell death regulator, is the key mediator of AQP1-initiated pro-tumor signaling involved in the pathophysiological process of TNBC.

RIPK1 represents an essential signaling node of cell death regulation in a variety of malignancies^24–27^. The current study revealed that RIPK1 is downregulated in a subtype-specific manner and is positively correlated with the prognosis of TNBC, but the correlation was not significant (*p* = 0.065) in our data, which may be attributed to the short follow-up interval or low patient numbers. Moreover, the abundance of RIPK1 is negatively correlated with that of AQP1 in our clinical cohorts, which inspired us to further explore the molecular relationship between them. The co-immunoprecipitation and mass spectra results confirmed that AQP1 binds to RIPK1 in TNBC. In addition, AQP1 and RIPK1 colocalize in the cytoplasm of TNBC tissue samples and cell lines, which paves the way to their mutual regulation. The in vitro results demonstrated that RIPK1 negatively regulates AQP1 signaling and TNBC cell proliferation, migration, and invasion. Besides, RIPK1 exhibited an inhibitive effect on the carcinogenic activity of AQP1 in vivo. The function of RIPK1 alone was not involved in the in vitro and in vivo study because the mechanism of AQP1-driven TNBC progression and metastasis is main aim of the current study, and RIPK1 was identified as a negative mediator. However, the regulatory role of AQP1 in TNBC-specific RIPK1 depletion remains unclear.

Canonically, RIPK1 can recruit RIPK3, which further phosphorylates the mixed lineage kinase domain–like pseudokinase (MLKL) to compose the necrosome, which is the core of the necroptosis machinery^15–18^. On the other hand, RIPK1 can bind to caspase-8 and FAS-associated death domain protein (FADD) to assemble the ripoptosome, resulting in the homodimerization and activation of caspase-8 to stimulate apoptosis^19–21^. Meanwhile, RIPK1 shares a key aspartate-specific residue (D324), which is the binding site of caspase-8 for RIPK1 cleavage, and plays an important role in termination of abnormal RIPK1 activation via the negative feedback mechanism^37,38^. The present study revealed both RIPK1/RIPK3/MLKL and RIPK1/caspase-8/caspase-3 pathways were inhibited in TNBC tumor samples and AQP1-expressing cell lines, which is in agreement with our presumption that both RIPK1-dependent necroptosis and apoptosis are attenuated by AQP1. Moreover, RIPK1^D324K^ mutant failed to be immunoprecipitated by AQP1, demonstrating D324 is the binding site of AQP1. The caspase-8-mediated cleavage and inactivation of RIPK1 were exacerbated in AQP1-expressiong cells, whereas RIPK1^D324K^ overexpression significantly prevented RIPK1 cleavage and weakened the aggressiveness of AQP1-enriched TNBC cells. Thereby it is reasonable that the combination of AQP1 with RIPK1 at D324 sequence is an obligatory step for TNBC to facilitate the recruitment of caspase-8, aggravate the cleavage of RIPK1, sequester RIPK1 in a quiescent form, and finally unleash tumor progression. It is argued that the deterioration of RIPK1 activity can promote caspase-8-FADD-induced apoptosis^39^, indicating that the cleavage and inactivation of RIPK1 may be unnecessary for AQP1-driven TNBC cell survival and migration. The claim, together with RIPK1-independent apoptosis, may just represent an idealistic paradigm when necroptosis is completely suppressed in cell cultures. Thus, RIPK1-dependent necroptosis and apoptosis have been identified as the main forms of cell death in the pathogenesis of multiple diseases^21,32,33,40^; however, in TNBC, the RIPK1-dependent cell death signals are arrested by AQP1 through excessively activating the caspase-8/RIPK1 negative feedback mechanism.

Consequently, the present work identifies a previously unrecognized pathway that RIPK1 binds to AQP1 and negatively mediates AQP1-driven TNBC progression and metastasis, and reciprocally, AQP1 adheres to and facilitates the cleavage of RIPK1 and suppresses the cell death signaling in TNBC. This initial foray will provide us a better understanding of the role of AQP1 in TNBC progression and create hope for potential therapeutic interventions (e.g., RIPK1 agonist by competitively inhibiting D324 activity) on the aggressive disease. However, further studies remain required to determine other components or adaptors of AQP1–RIPK1 complex, the mechanism of caspase-8 recruitment by AQP1 after binding to RIPK1 and the intrinsic influence of the novel pathway on the microenvironment of TNBC.

## Methods

### Antibodies and reagents

Mouse monoclonal antibodies against AQP1 (ab9566) and RIPK1 (ab72139) were purchased from Abcam (Cambridge, MA). Mouse monoclonal antibodies against RIPK1 (610458), caspase-8 (9746), and RIPK3 (sc-374639) were bought from BD Biosciences (San Jose, CA), Cell Signaling Technology (Danvers, MA), and Santa Cruz Biotechnology (Dallas, TX), respectively. The rabbit monoclonal antibody against p-MLKL (S345) (ab196436), p-RIPK3 (S227) (ab209384) and p-RIPK3 (S232) (ab195117), and rabbit polyclonal antibody against RIPK1 (ab106393) and MLKL (ab194699) were purchased from Abcam. The rabbit polyclonal antibody against cleaved caspase-3 (9661) and caspase-3 (9662) were bought from Cell Signaling Technology. The rabbit monoclonal anti-β-actin antibody (AC026) and anti-α-tubulin antibody (AC013) were from Abclonal (Wuhan, China). The cell transfection regent (F231-02) was purchased from TransGen Biotech (Beijing, China). The point mutation plasmids of RIPK1 were constructed using the HiFi HotStart DNA Polymerase (KAPA Biosystems, Roche, Switzerland).

### In silico data analysis

The log2-transformed gene expression profiles of AQP1 and RIPK1 in invasive breast carcinoma were retrieved from the Finak’s and Curtis’s cohorts in the Oncomine database (www.oncomine.org). TNBC datasets were downloaded from TCGA, GTEx, and the gene expression omnibus data repository (GEO)^41–43^. Additional information was extracted from the corresponding literatures and supplementary data. The expression levels of AQP1 and RIPK1 were normalized, log2 transformed, and compared using RMA and MAS version 5.0.

### Patient tissue sample collection

The clinical tissue samples were obtained from 62 unilateral TNBC patients between May 2012 and October 2018 at Tianjin Medical University Cancer Institute and Hospital and the First Affiliated Hospital of Xiamen University. The study conformed to the Ethical Guidelines of the Helsinki Declaration, and was approved by the Ethics Committee of Tianjin Medical University Cancer Institute and Hospital, Tianjin, and the Ethics Committee of the First Affiliated Hospital of Xiamen University, Xiamen, People’s Republic of China. After given the written informed consent, the patients underwent either breast conserving or ablative surgery. The excess tumor and normal breast tissue specimens were collected. The cases pathologically diagnosed as TNBC without any neoadjuvent anticancer therapy were involved in the current study. The clinical specimens were divided into two pieces: one was immediately frozen by liquid nitrogen and stored at −80 °C; the other was fixed with 4% formaldehyde for paraffin embedding.

### Hematoxylin–eosin and immunohistochemistry staining

The paraffin-embedded samples were cut into longitudinal sections of 5 μm, which were stained by hematoxylin–eosin and observed under a light microscope. The immunohistochemistry assessment for AQP1 and RIPK1 was conducted using standard techniques by streptavidin–peroxidase method. The antigen retrieval was performed in sodium citrate (1 mM, pH 6.0) using an autoclave (120 °C for 2 minutes). After serial blocking with 3% H_2_O_2_ and 10% normal goat serum, the sections were incubated with anti-AQP1 (diluted 1:500) or anti-RIPK1 (diluted 1:400) antibody at 4 °C overnight. The sections were then incubated with biotinylated goat anti-rabbit immunoglobulin, and the reaction was visualized with the DAB complex followed by incubation with HRP–streptavidin. The results were assessed by a single pathologist in a double-blind manner. The positivity was defined as the score ≥3 according to the previous scoring system, using staining intensity multiplied by staining area^30^. The percentage of positive cells per high-power field (HPF) was counted, and the average values of 5-HPF percentage counts were involved into the final calculation.

### Mass spectrometry

For LC–MS/MS analysis, we separated peptides using a Thermo-Dionex Ultimate 3000 HPLC system. The analytical column was a homemade fused silica capillary column (75 μm ID, 150 mm length; Upchurch, Oak Harbor, WA) packed with C-18 resin (300 A, 5 μm; Varian, Lexington, MA). Each LC–MS/MS run were searched against the AQP1 and RIPK1, using Proteome Discoverer (Version PD1.4, Thermo Fisher Scientific, USA) searching algorithm. Peptides assigned to a given protein group were considered as unique and more than two unique peptide matches was quantified.

### Cell culture, lentiviral infection, and plasmid transfection

The MDA-MB-231 cells (human TNBC cells, no. 3111C0001CCC000014) were obtained from the Cell Resource Center of Institute of Basic Medicine, Chinese Academy of Medical Sciences (Beijing, China). The HEK-293T cells (SV40T transformed human embryonic kidney cells, no. 3131C0001000200017) and 4T1 cells (mouse TNBC cells, no. 3131C0001000800032) were purchased from the Cell Resource Center, Shanghai Institutes for Biological Sciences, Chinese Academy of Sciences (Shanghai, China). They were cultured in the Dulbecco’s modified Eagles medium (DMEM; HyClone, South Logan, UT) supplemented with 10% fetal bovine serum (FBS; Life Technologies, Carlsbad, CA), 100 U/ml penicillinm, and 100 μg/ml streptomycin (Life Technologies, Carlsbad, CA) in a 5% CO_2_ incubator at 37 °C.

Stable overexpressing cell lines were established using a lentiviral system. The pCDH-CMV lentiviral vector expressing human AQP1 (NM_198098) ORF in conjunction with a tag of 3×FLAG and a puromycin selection marker was established, while the pCDH-RIPK1 (NM_003804)-HA and pCDH-RIPK1^D324K^-HA with a G418 selection marker were obtained, following the manufacturer’s instructions (Hanbio, Shanghai, China). Lentiviruses were generated by co-transfection into HEK-293T cells with vectors or plasmids at 2 µg/ml, pMD2.G envelope (no. 12259, AddGene, Watertown, MA) and psPAX2 (no. 12260, AddGene, Watertown, MA) packaging vectors. The supernatants containing viral particles were collected to infect the TNBC cells at a multiplicity of infection of 25. Stable clones were screened out via puromycin or G418 selection at 1 µg/ml.

To temporally knockdown the RIPK1 expression, TNBC cells at ~80% confluence were transfected with 200 pmol control siRNA or siRNA targeting RIPK1, according to the manufacturer’s instruction (TransGen Biotech, Beijing, China). The siRNA against human RIPK1 and Scrambled control siRNA were established, using previously described methodology^27^. Mouse RIPK1-siRNA duplexes (SR418761) and Scrambled Negative Control siRNA duplex (SR30004) were purchased from OriGene (Rockville, MD).

Point mutation plasmids of RIPK1 were established, as previously described^27^. In brief, the PCR-amplified products were digested with Dpn1 restriction enzyme (NEB, Ipswich, MA, USA) and transformed into TransT1 cells (TransGen Biotech, Beijing, China). The plasmid DNAs were extracted from different cell colonies and verified by sequencing. The plasmids were then transformed into HEK-293T cells together with the AQP1-overexpressing plasmid with a tag of 3×FLAG. The interaction of AQP1 and RIPK1 were further detected by co-immunoprecipitaion and subsequent Western blot analysis.

### Cell proliferation, migration, and invasion assays

The TNBC cell proliferation was assessed by measuring the absorbance at 450 nm wavelength using a CCK-8 (Dojindo, Kumamoto, Japan) under the direction of the manufacturer’s instruction after incubation at 37 °C for 1 hour. The absorbance was detected every 24 hours for 3 days. The cell viability was defined as (OD_treatment group_ − OD_blank_)/(OD_standard control_ − OD_blank_) × 100%.

The wound healing assay was applied to analyze the TNBC cell migration. Briefly, 5 × 10^5^ cells were seeded in six-well plates and incubated overnight in DMEM supplemented with 10% FBS. Wound injury was made using the tip of a sterile micropipette and the cells were allowed to migrate for up to 24 hours in serum-free media. The images of the wounds were obtained from five random microscopic fields in three independent wells. The width of the wound gaps were measured by Image J software (National Institutes of Health, Bethesda, MD) to calculate the wound healing percentage.

In vitro migration and invasion assays were performed using a 24-well transwell insert (8 μm pore size, Corning, Corning, NY). The upper chambers were precoated with Matrigel matrix (Corning, 356234; Thermo Fisher Scientific, Pittsburgh, PA) for the invasion assays. A total of 1 × 10^5^ TNBC cells in 100 μl serum-free media were plated on the upper chambers, and 500 μl of DMEM supplemented with 20% FBS was filled in the lower chambers. Then, the cells were allowed to migrate or invade for 20 h, fixed with 4% formaldehyde, stained with 0.5% crystal violet, and rinsed by PBS. The chambers per condition were imaged in three independent tests with five random microscopic fields per chamber. The number of migrated or invaded cells were counted and analyzed by Image J software.

### Western blot analysis

The tumors and cells were homogenized in the RIPA lysis buffer, containing protease inhibitor cocktail (Roche Diagnostics, Mannheim, Germany). Total protein was separated by sodium dodecyl sulfate–polyacrylamide gel electrophoresis. Proteins were blotted to polyvinylidene difluoride membranes (Amersham Biosciences, Arlington Heights, IL). Blots were incubated with primary antibodies (diluted 1:1000 except for β-actin antibody [diluted 1:3000] and α-tubulin antibody [diluted 1:5000]) at 4 °C overnight, respectively. HRP-labeled secondary antibodies (Zhongshan, Beijing, China) were then added, and the blots were developed with the ECL plus kit (Amersham Biosciences). The images from the same experiments were scanned, and the gray values were processed and analyzed in parallel by Image J software.

### Immunoprecipitation

The tumor tissues or cells were prepared in the RIPA lysis buffer (Roche Diagnostics, Mannheim, Germany). The lysates were precleaned with protein G agarose beads (Thermo Fisher Scientific, Waltham, MA, USA) at 4 °C for 4 h. Control IgG, anti-RIPK1 antibody (diluted 1:200), anti-AQP1 antibody (diluted 1:400), or anti-FLAG antibody (diluted 1:200) was coupled to protein G agarose beads in lysis buffer for 4 hours at 4 °C. Then, the lysates were incubated with beads coupled with different antibodies or control IgG overnight at 4 °C. The complexes were precipitated and collected for Western blot analysis.

### Immunofluorescence staining

The colocalization of AQP1 and RIPK1 was examined in clinical specimens and TNBC cells. MDA-MB-231 and 4T1 cells stably overexpressing both AQP1 and RIPK1 were cultured in glass-bottom dishes (Nunc; Thermo Fisher Scientific, Waltham, MA, USA). Cells were washed with PBS, fixed with 4% formaldehyde, and permeabilized by Triton X-100. The cells and tissue samples were incubated with anti-AQP1 (diluted 1:400) and anti-RIPK1 (diluted 1:200) antibodies at 4 °C overnight, and then with goat anti-mouse antibody (green fluorescence, ab150117, Abcam, Cambridge, MA) and donkey anti-rabbit antibody (red fluorescence, ab150075, Abcam) at room temperature for 1 h. DAPI was used to stain the cell nuclei. The images of staining were captured by a Zeiss LSM800 confocal microscope.

### In vivo orthotopic breast carcinoma model and experiments

All animal care and studies were performed with the approval of the Ethics Committee of our institution and hospital. The orthotopic breast carcinoma mouse model was constructed, as previously described^44^. Briefly, the transgenic 4T1 cells (1 × 10^5^/mouse) were orthotopically injected into the mammary fat pad of 7-week-old female BALB/c mice (Charles River Laboratories, Beijing, China) using a 1-ml tuberculin syringe. The tumor length and width were measured with vernier calipers every 7 days in the first 2 weeks after implantation, and every 3 days in the remaining time until the end of observation. The tumor volume was defined using the formula *V* = (*L* × *W* × *W*)/2, where *V* represents tumor volume, *L* refers to tumor length, and *W* equals to tumor width.

For spontaneous lung metastasis detection, six mice per group were sacrificed at 32^nd^ day after surgery. The tumors were obtained to precisely measure the diameters. The lungs were excised, fixed in formalin, paraffin embedded, and sectioned for hematoxylin–eosin staining. The metastatic colonies of each lung sample section were photographed under a microscope at ×4 magnification. The metastatic lesion area from five random microscopic fields were measured using Image J software. In addition, the overall survival rate was estimated by Kaplan–Meier curve and subsequent log-rank analysis in consecutive observations of 33 mice (11 per group) for 105 days.

### Statistical analysis

All results are presented as mean ± SD (standard deviation). Statistical analysis was performed using two-tailed paired and unpaired Student’s *t* test, one-way analysis of variance with Bonferroni post hoc analysis and Pearson’s correlation test, using GraphPad Prism version 8.01 and SPSS version 20.0. Survival curves were generated using the Kaplan–Meier product-limit method and compared with the log-rank test. Significance was assumed with a *p* < 0.05.

### Reporting summary

Further information on research design is available in the Nature Research Reporting Summary linked to this article.

## Supplementary information

Supplementary Information

Reporting Summary

## Data Availability

The data generated and analyzed during this study are described in the following data record: 10.6084/m9.figshare.14394161 (ref. ^45^). The public data resources used in the related study are openly available from the following sources: the Oncomine database (http://www.oncomine.org), the Cancer Genome Atlas (TCGA, https://identifiers.org/cbioportal:brca_tcga), Genotype-Tissue Expression (GTEx, https://gtexportal.org), and the Gene Expression Omnibus data repository (GEO, https://identifiers.org/geo:GSE1456, https://identifiers.org/geo:GSE6532, and https://identifiers.org/geo:GSE7390). The majority of the GraphPad Prism files underlying the figures and supplementary figures of the related article are openly available as part of this data record. However, several are saved in institutional storage and are not publicly available to protect the patient privacy. These may be available from the corresponding author upon reasonable request. All the uncropped Western blots generated during this study are available in Supplementary Fig. 6.
