# Supplementary material for: RIPK1 is a negative mediator in Aquaporin 1-driven triple-negative breast carcinoma progression and metastasis
Source: NPJ Breast Cancer. 2021 May 12;7:53. doi: 10.1038/s41523-021-00261-5 (PMC8115349; doi:10.1038/s41523-021-00261-5)

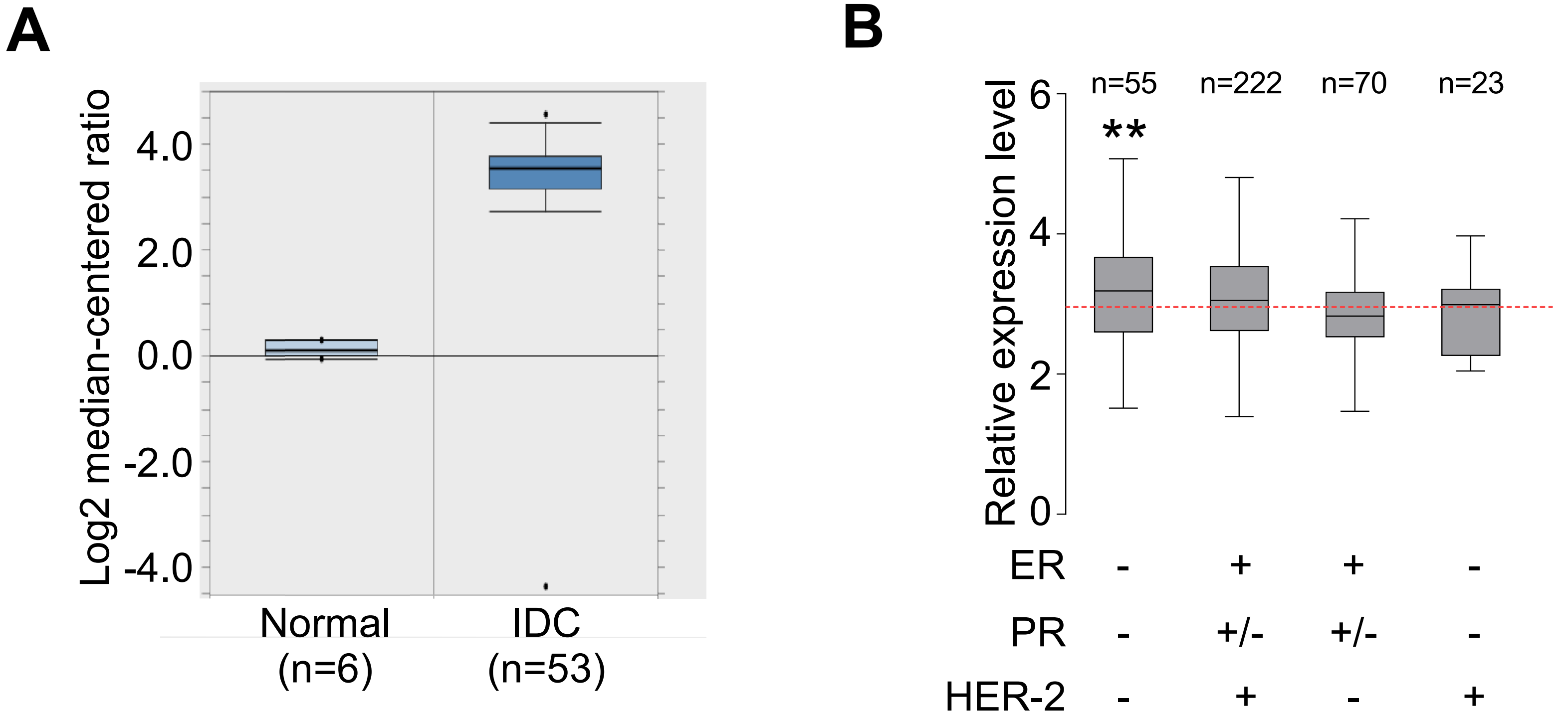

Supplementary Figure 1. Bioinformatics analysis showed AQP1 is upregulated in TNBC.

(A) Quantification of the Log2 transformed expression level of AQP1 in invasive ductal breast carcinoma (IDC) compared with normal breast tissue. Data were extracted from the Finak's cohorts in the Oncomine database. (B) Subtype-specific expression intensity of AQP1 in breast carcinoma. Data were retrieved from the TCGA and GTEx datasets (GSE1456/GSE6532/GSE7390). Note: ** $p < 0.01$.

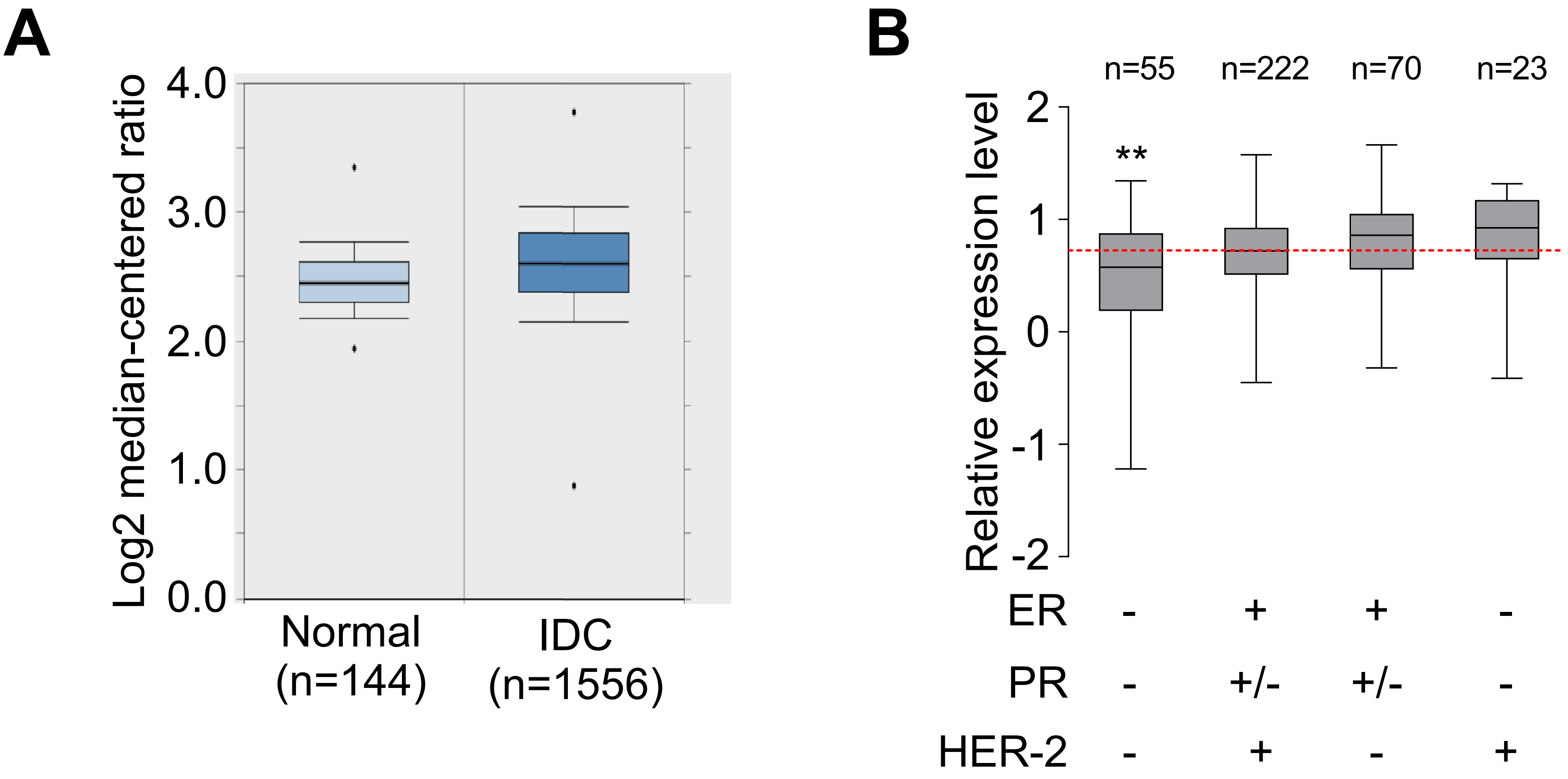

Supplementary Figure 2. Bioinformatics analysis showed RIPK1 is downregulated in TNBC.
(A) Quantification of the Log2 transformed expression level of RIPK1 in invasive ductal breast carcinoma (IDC) compared with normal breast tissue. Data were extracted from the Curtis's cohorts in the Oncomine database. (B) Subtype-specific expression intensity of RIPK1 in breast carcinoma. Data were retrieved from the TCGA and GEO datasets (GSE1456/GSE6532/GSE7390). Note: ** $p < 0.01$.

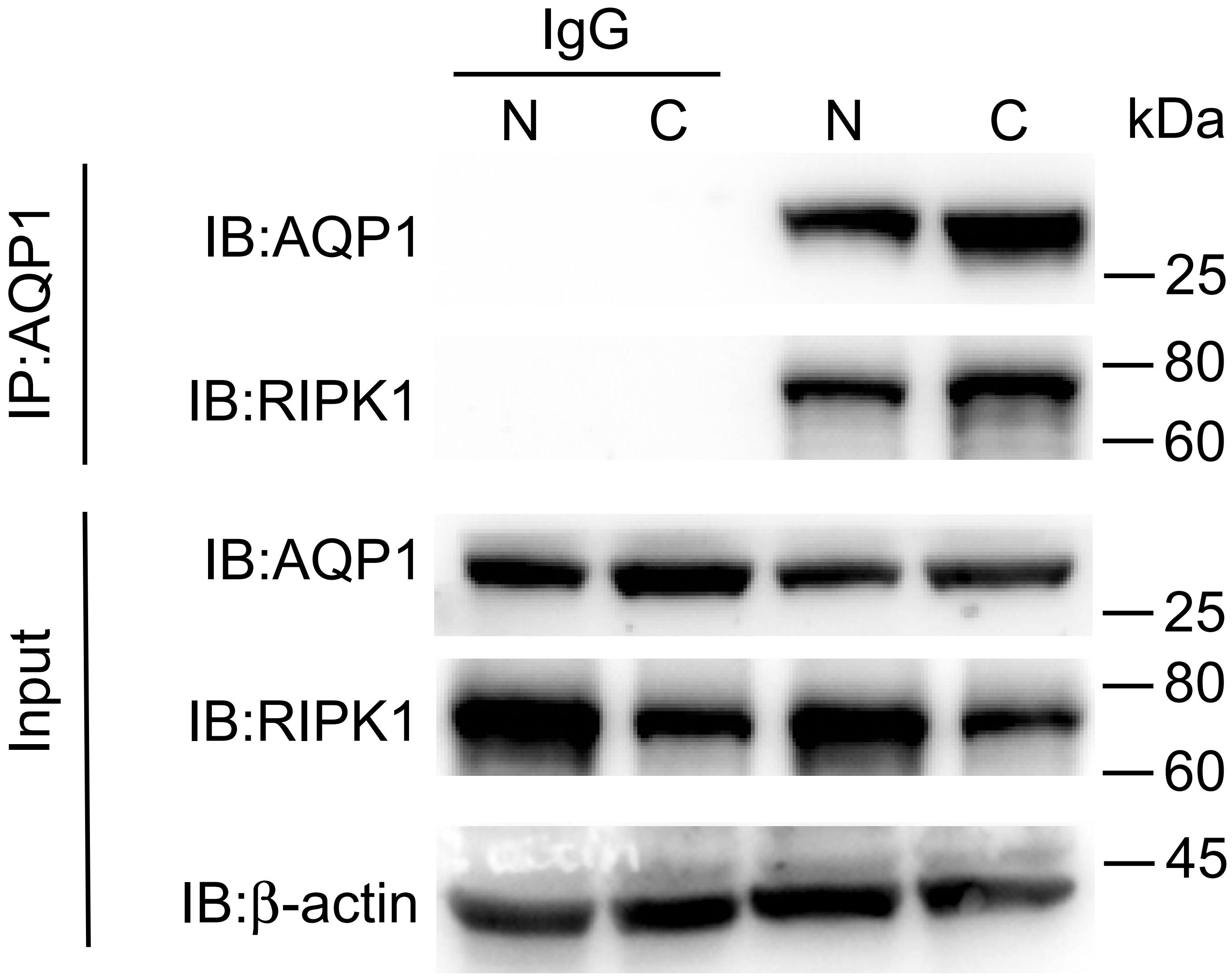

Supplementary Figure 3. Co-immunoprecipitation of AQP1 from TNBC and normal breast tissue showing RIPK1 binds with AQP1. *Abbreviations*: N, normal breast tissue; C, TNBC.

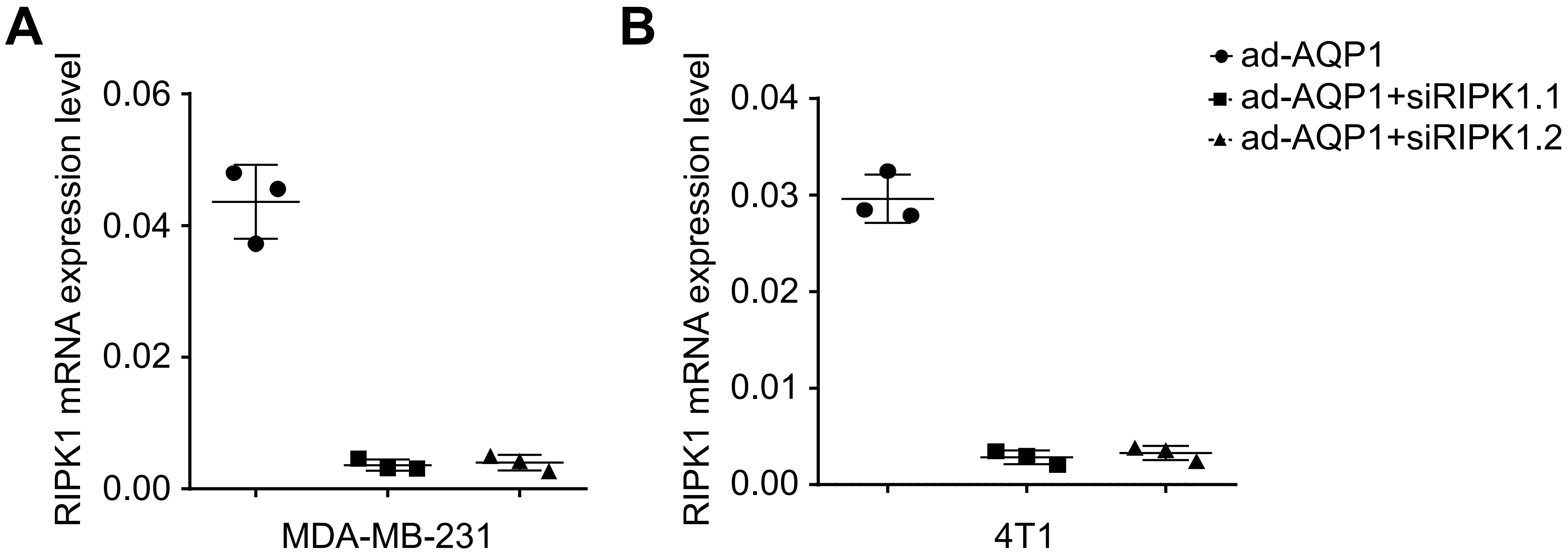

Supplementary Figure 4. Statistical analysis of the efficiency of RIPK1 siRNAs.
Both siRNA candidates demonstrated over 90% decrease of the abundance of RIPK1 mRNA in MDA-MB-231 (n = 3) (A) and 4T1 cells (n = 3) (B).

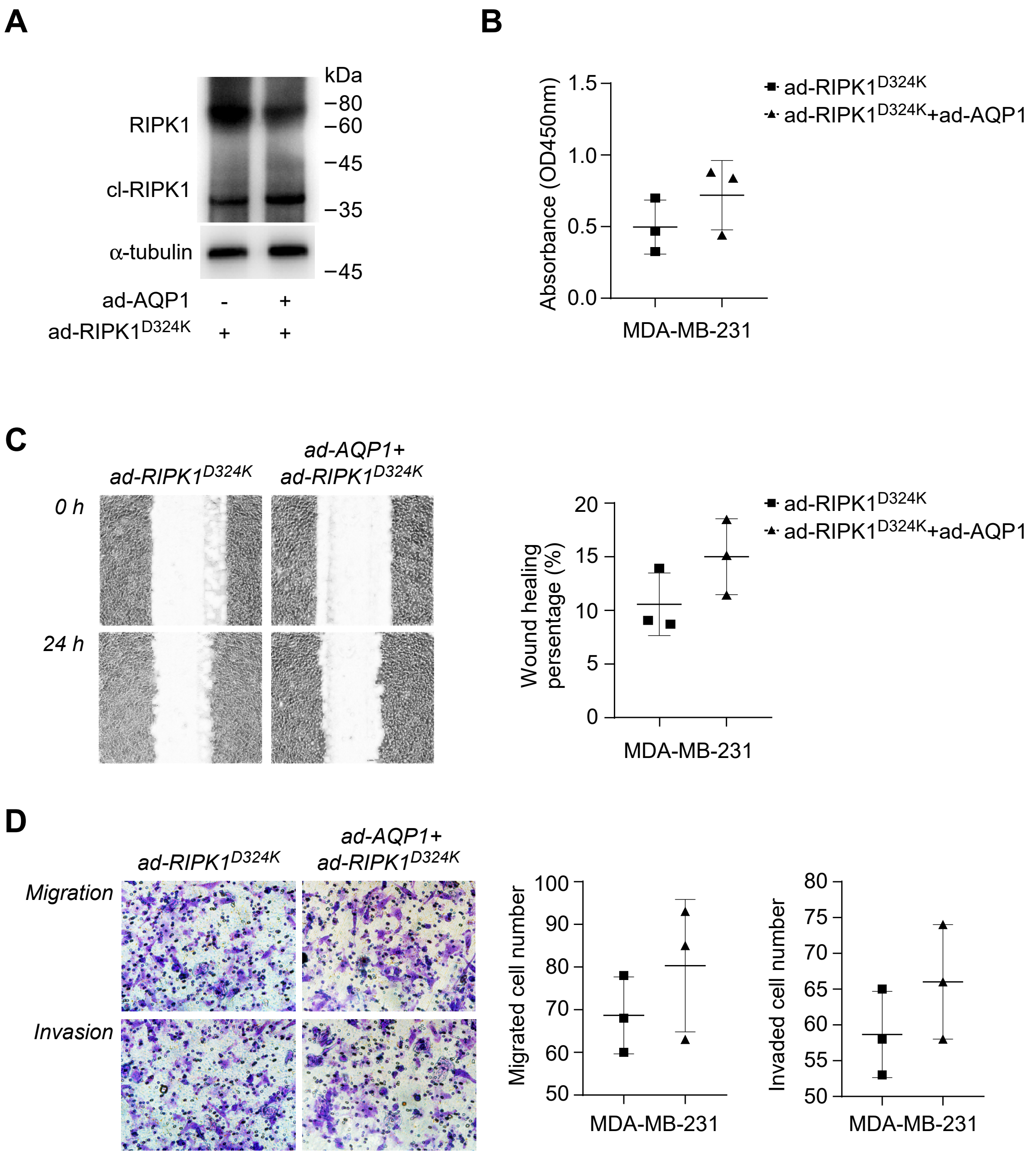

Supplementary Figure 5. RIPK1D324K completely inhibited AQP1-driven TNBC progression and metastasis in *vitro*.

(A) Western blot analysis of RIPK1 and cleaved RIPK1 (cl-RIPK1) in MDA-MB-231 cells stably overexpressing $RIPK1^{D324K}$ and AQP1. (B) Relative viability of MDA-MB-231 cells at 72 hours after cell seeding. The cell viability is displayed by the absorbance at 450 nm wavelength in the CCK-8 assay (n = 3). (C) Representative images and quantification of MDA-MB-231 cells in the wound-healing assay at 0 and 24 hours after micropipette scratching (X100 magnification, n = 3). (D) Representative images and quantification of MDA-MB-231 cells in the transwell migration and invasion assays (X400 magnification, n = 3). *Abbreviations*: ad-AQP1, AQP1 overexpression; ad-$RIPK1^{D324K}$, $RIPK1^{D324K}$ overexpression.

Supplementary Table 1. RIPK1 interacted with AQP1 by mass spectrum analysis.

Accession	Description	Score	Coverage	Proteins	Unique Peptides
P29972	Aquaporin-1 OS=Homo sapiens GN=AQP1 PE=1 SV=3 - [AQP1_HUMAN]	9.71	10.78	2	2
	Binding sequence [AQP1_HUMAN]	MASEFKKKLFWRAVVAEFLATTLFVFISIGSALGFKYPVGNNQTAVQDN VKVSLAFGLSIATLAQSVGHISGAHLNPAVTLGLLLSCQISIFRALMYIIA QCVGAIVATAILSGITSSLTGNSLGRNDLADGVNSGQGLGIEIIGTLQLVL CVLATTDRRRRDLGGSAPLAIGLSVALGHLLAIDYTGCGINPARSFGSAV ITHNFSNHWIFWVGPFIGGALAVLIYDFILAPRSSDLTDRVKVWTSGQVE EYDLDADDINSRVEMKPK			

Uncropped blots

Figure 1C

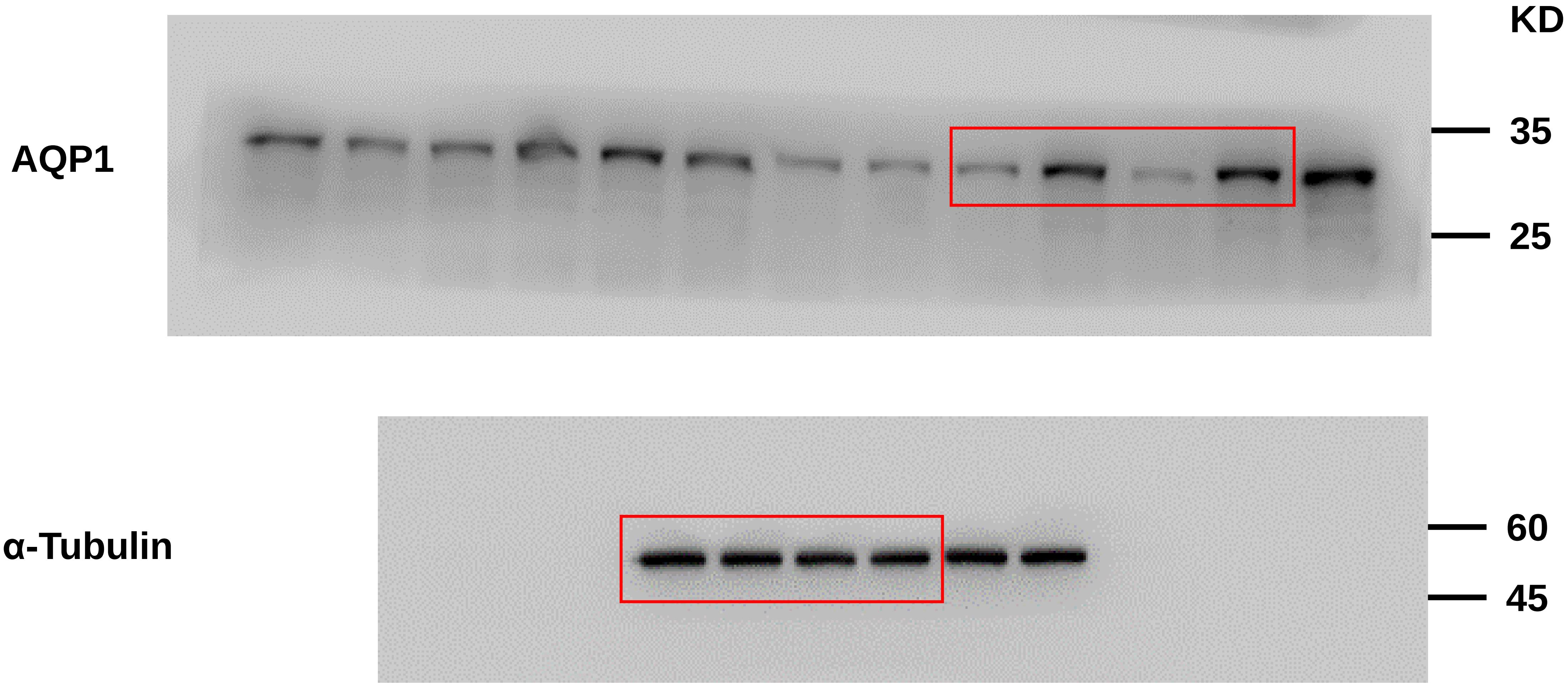

Figure 2C

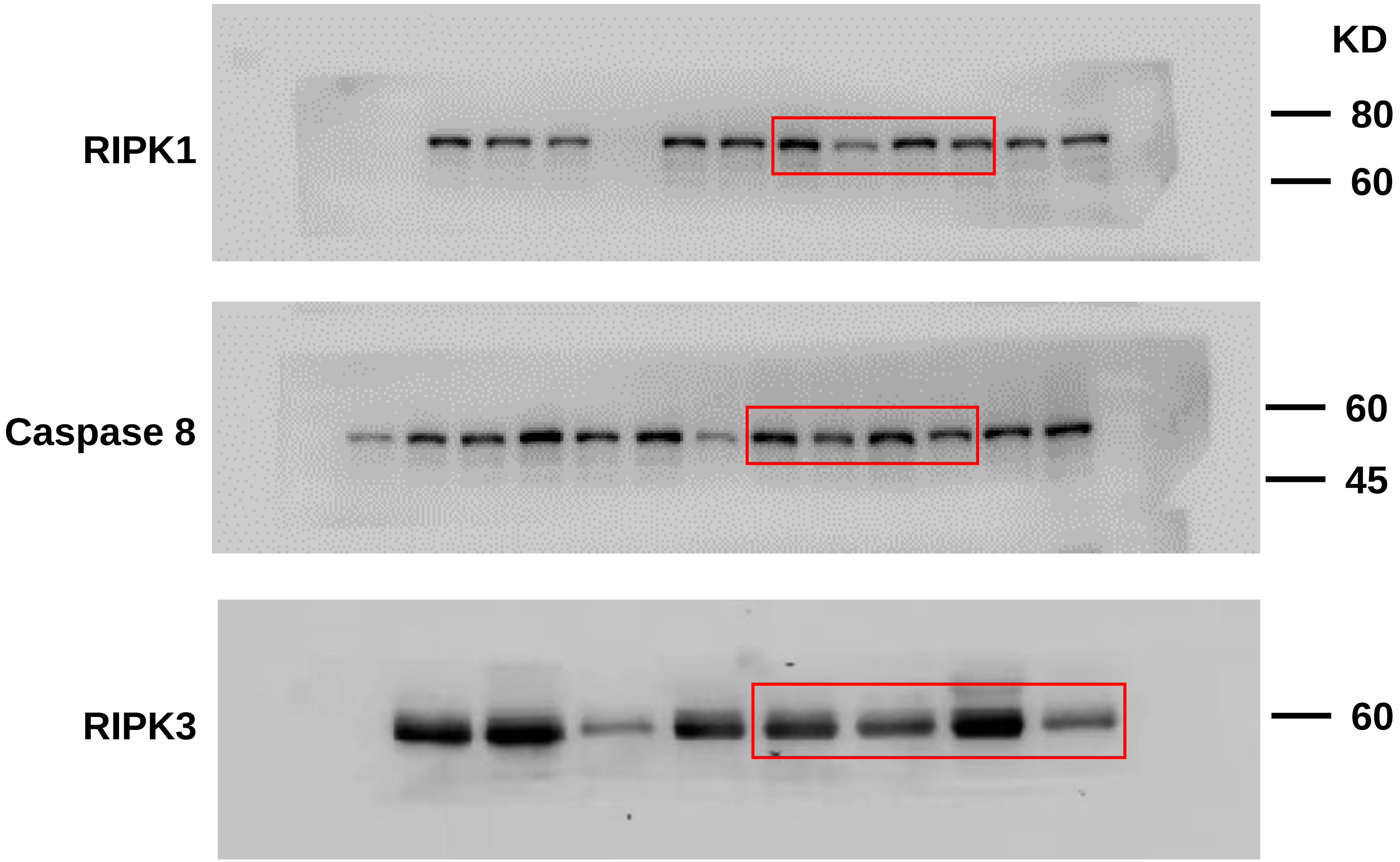

Figure 2C

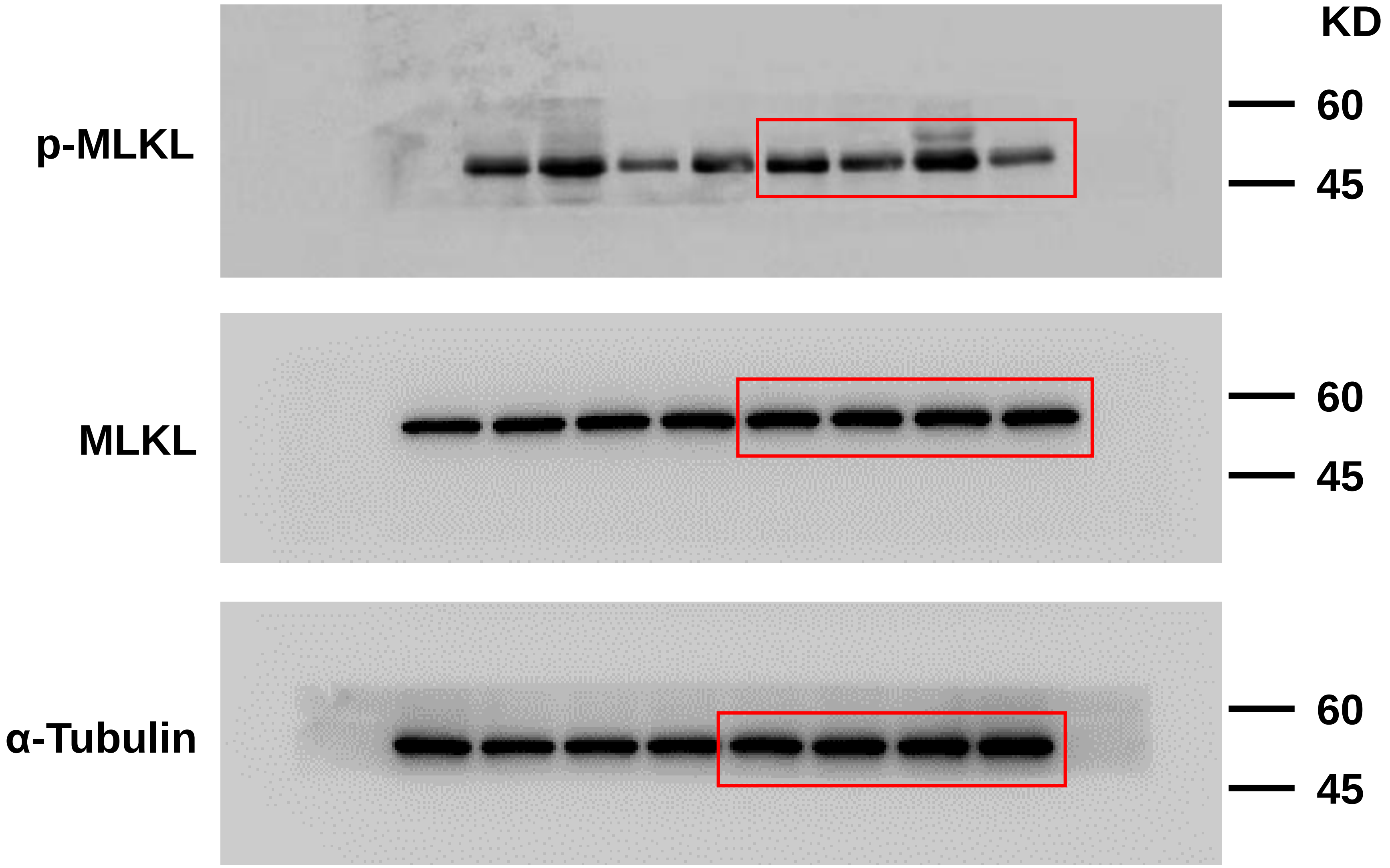

Figure 3B

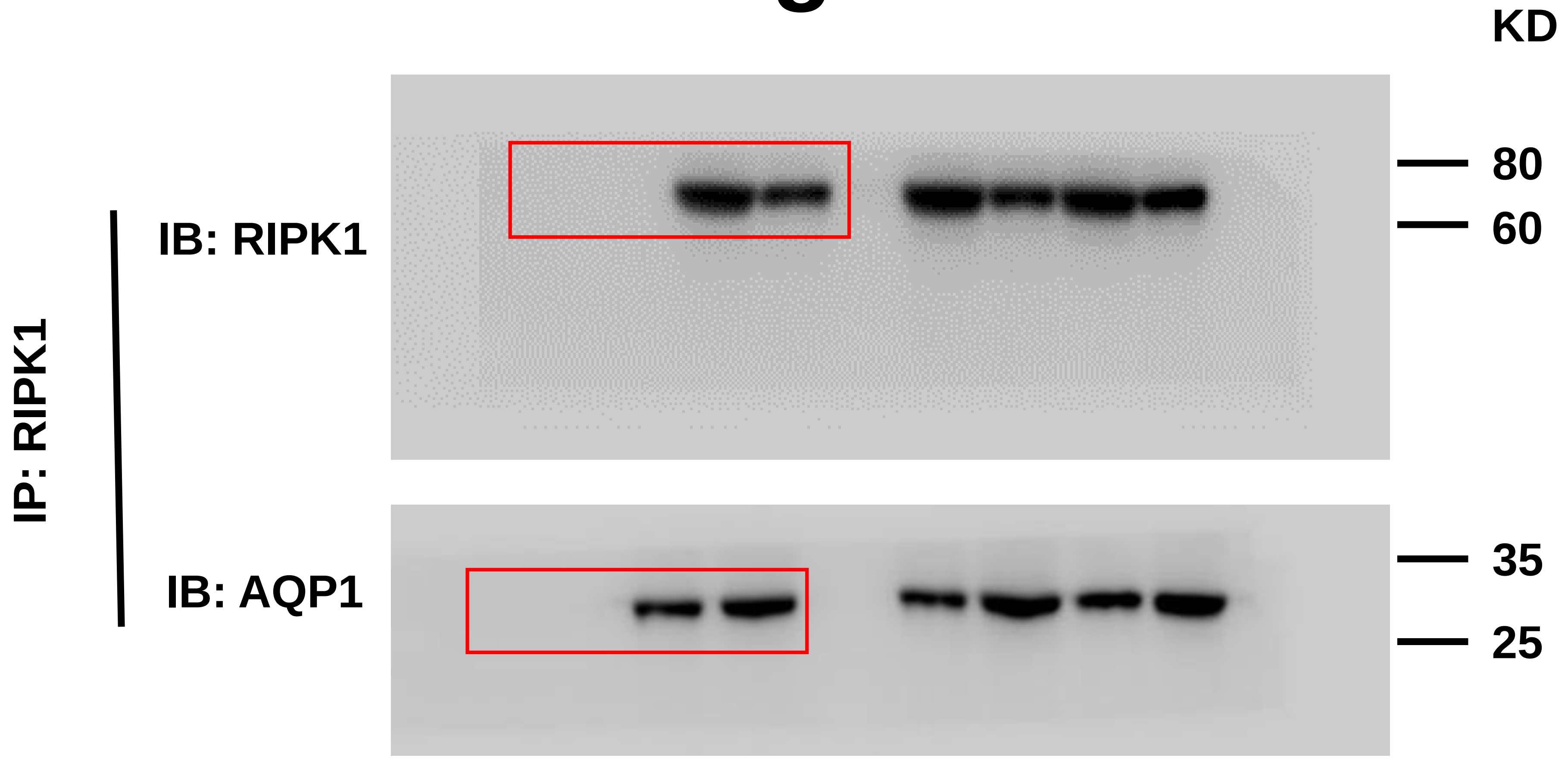

Figure 3B

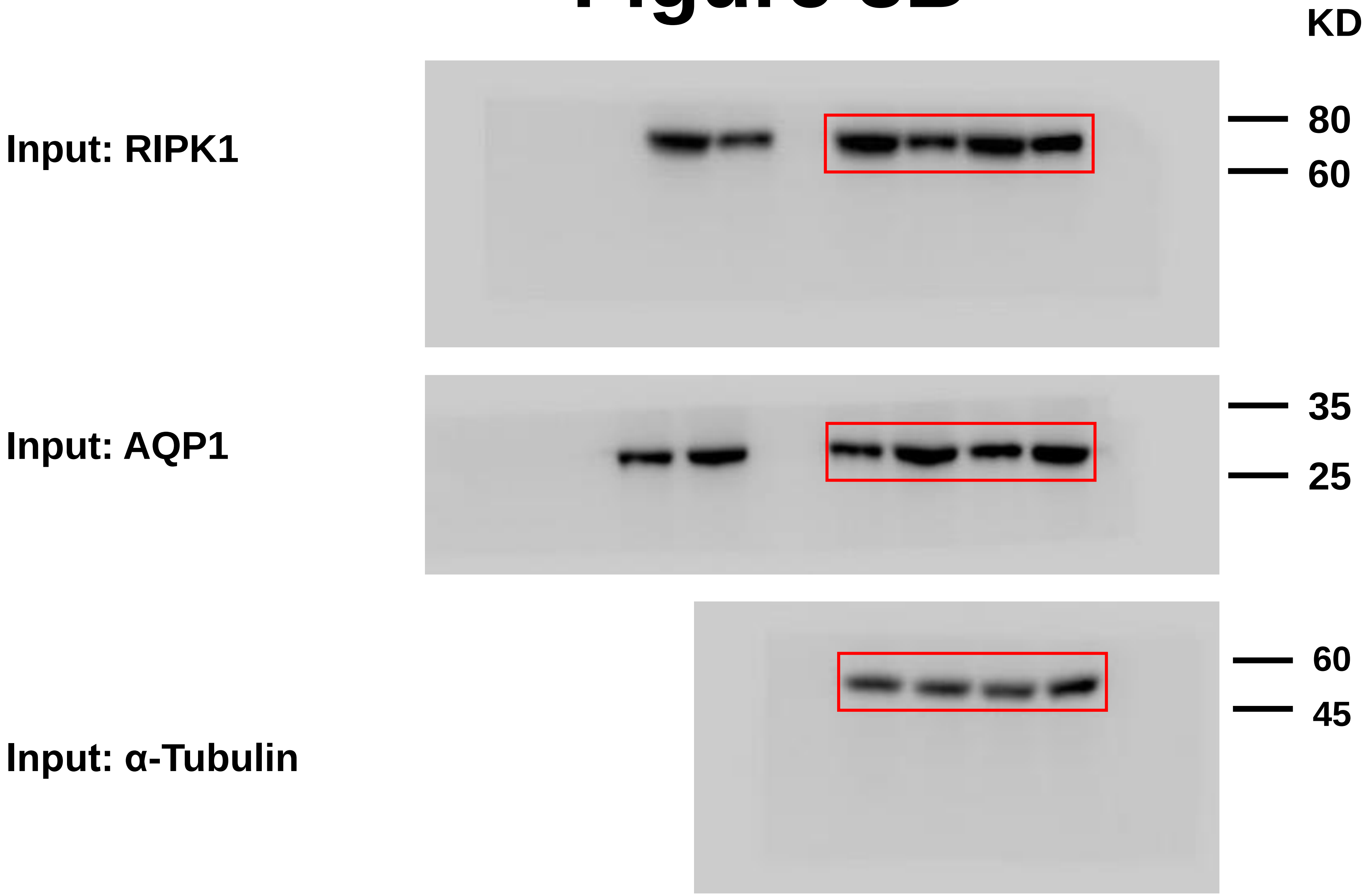

Figure 4A- MDA-MB-231

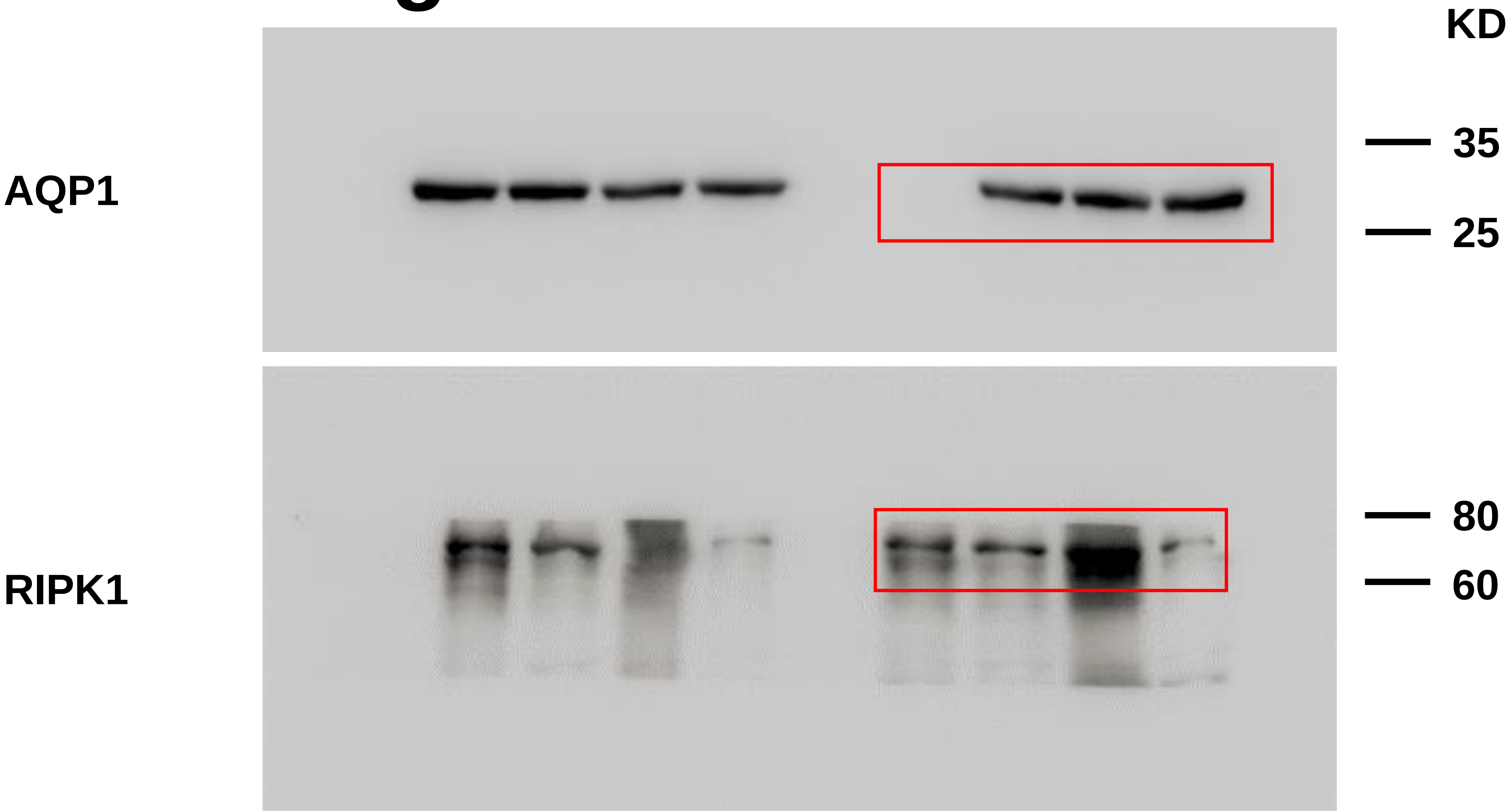

Figure 4A- MDA-MB-231

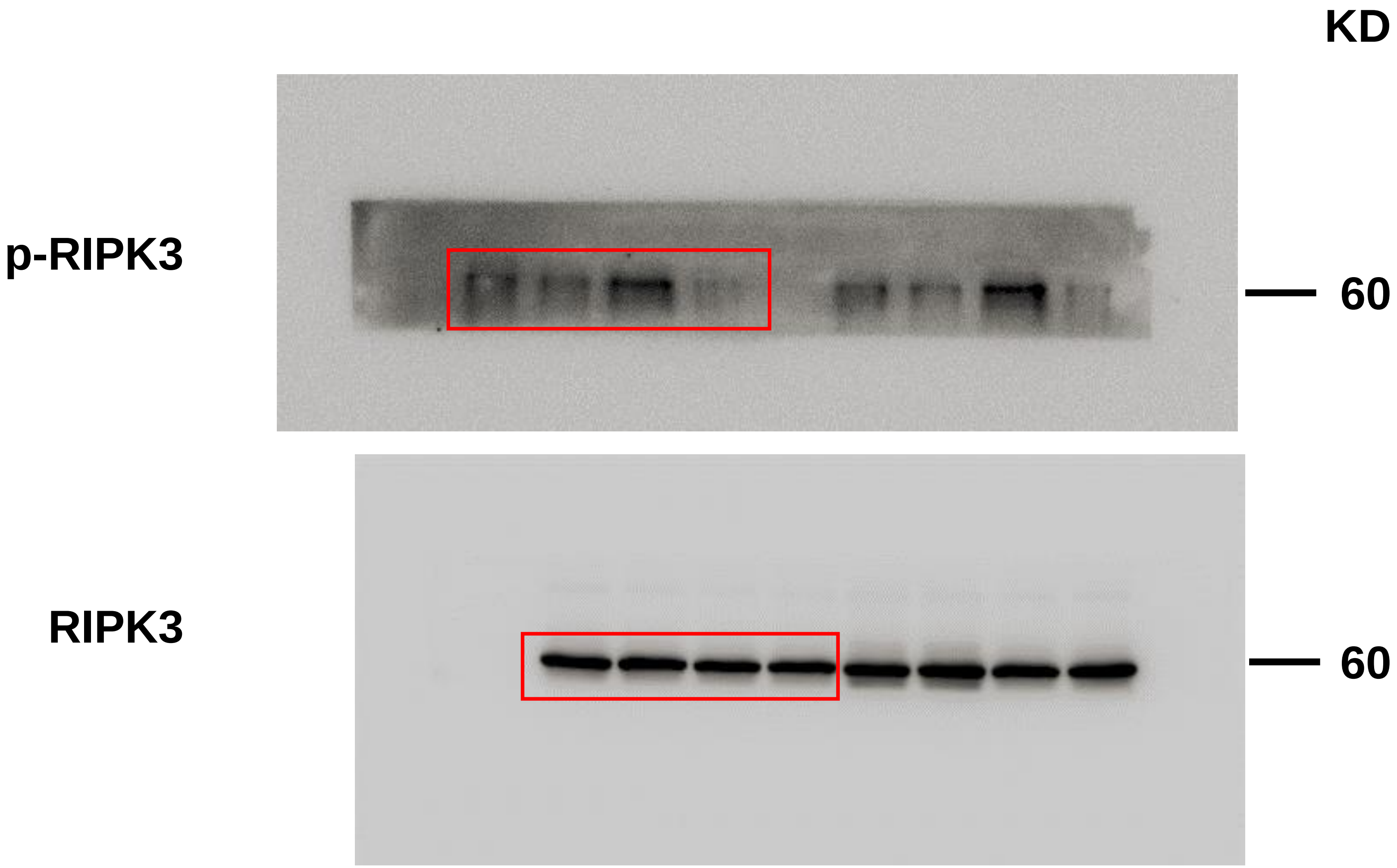

Figure 4A- MDA-MB-231

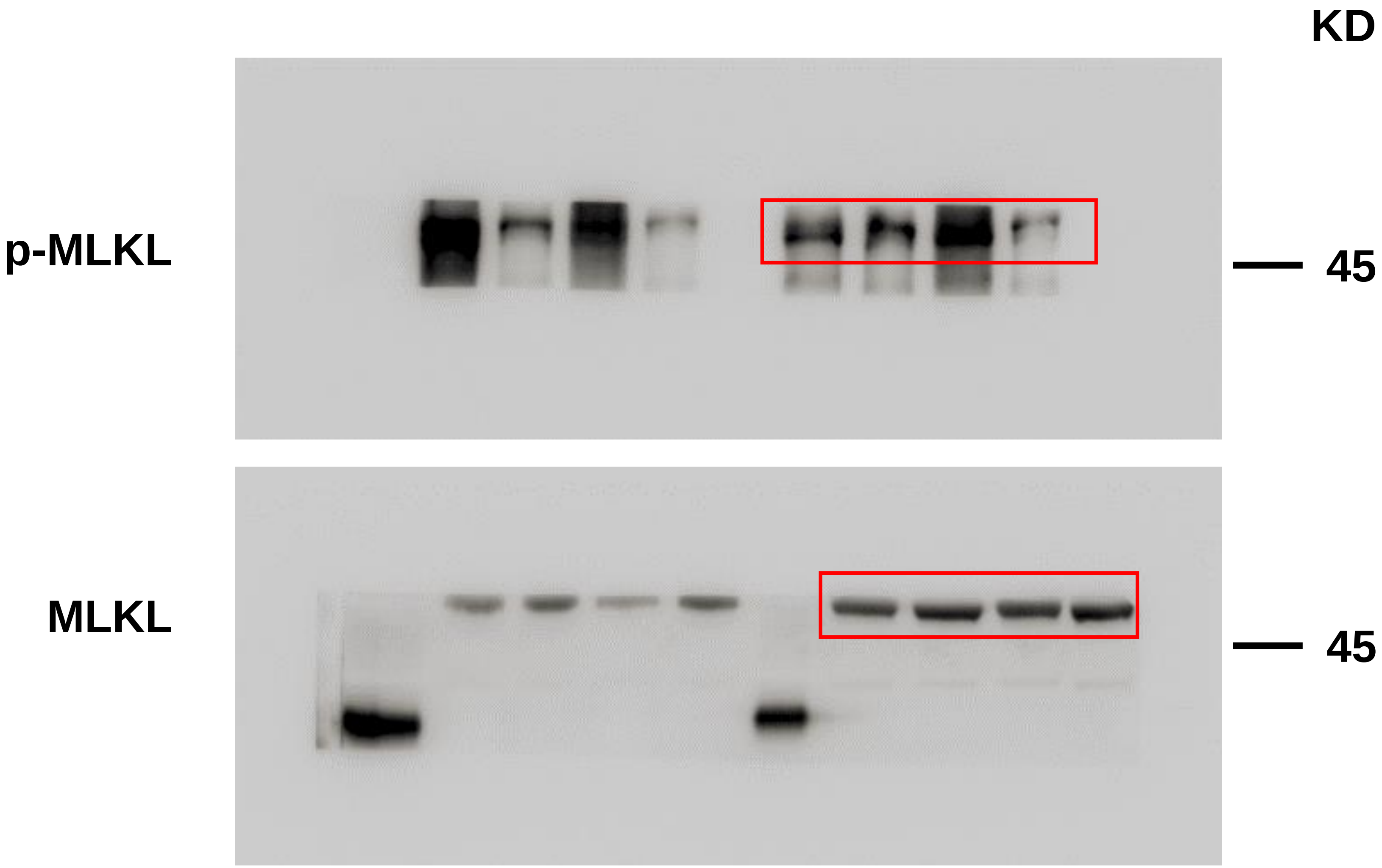

Figure 4A- MDA-MB-231

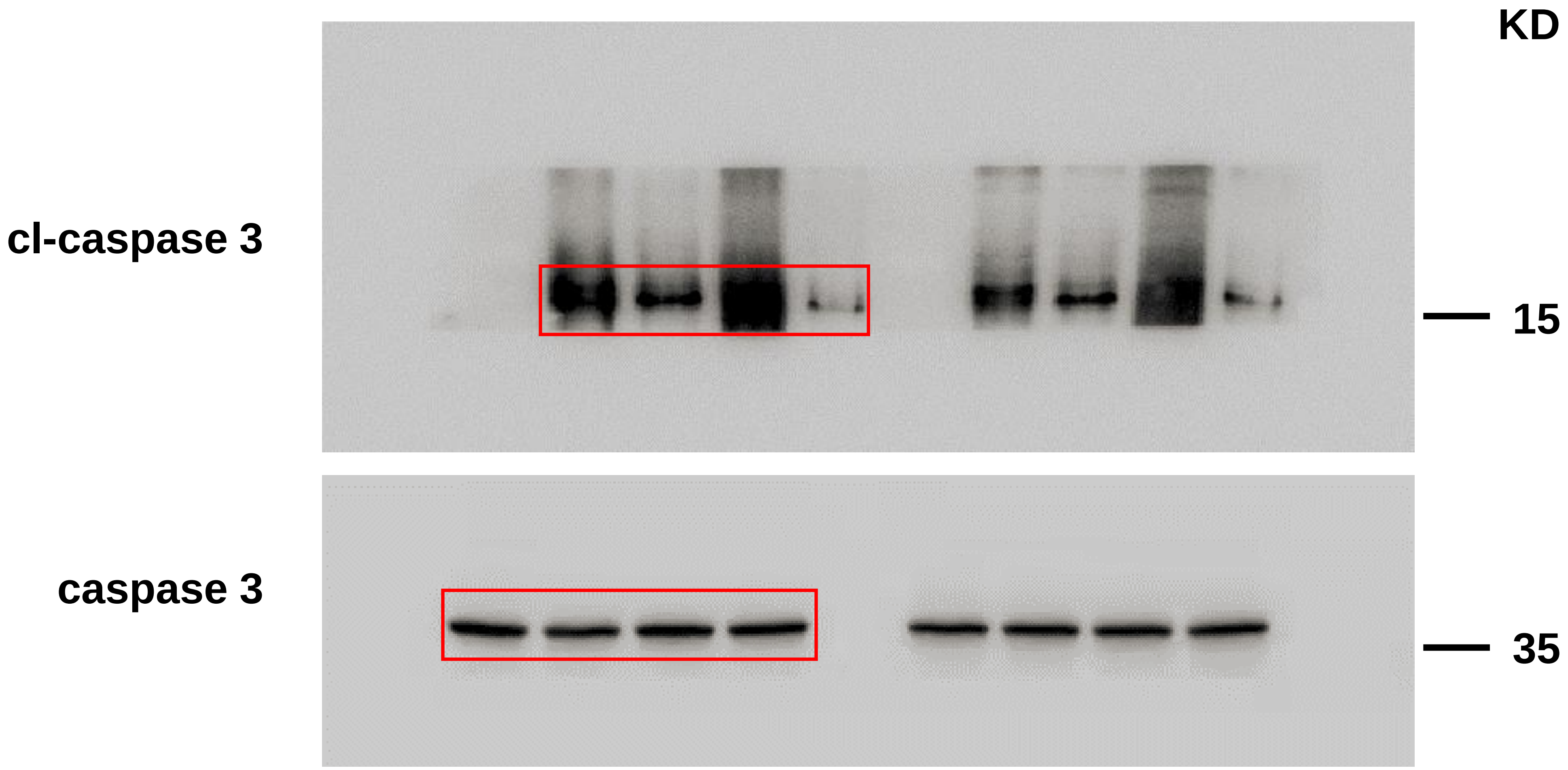

Figure 4A- 4T1

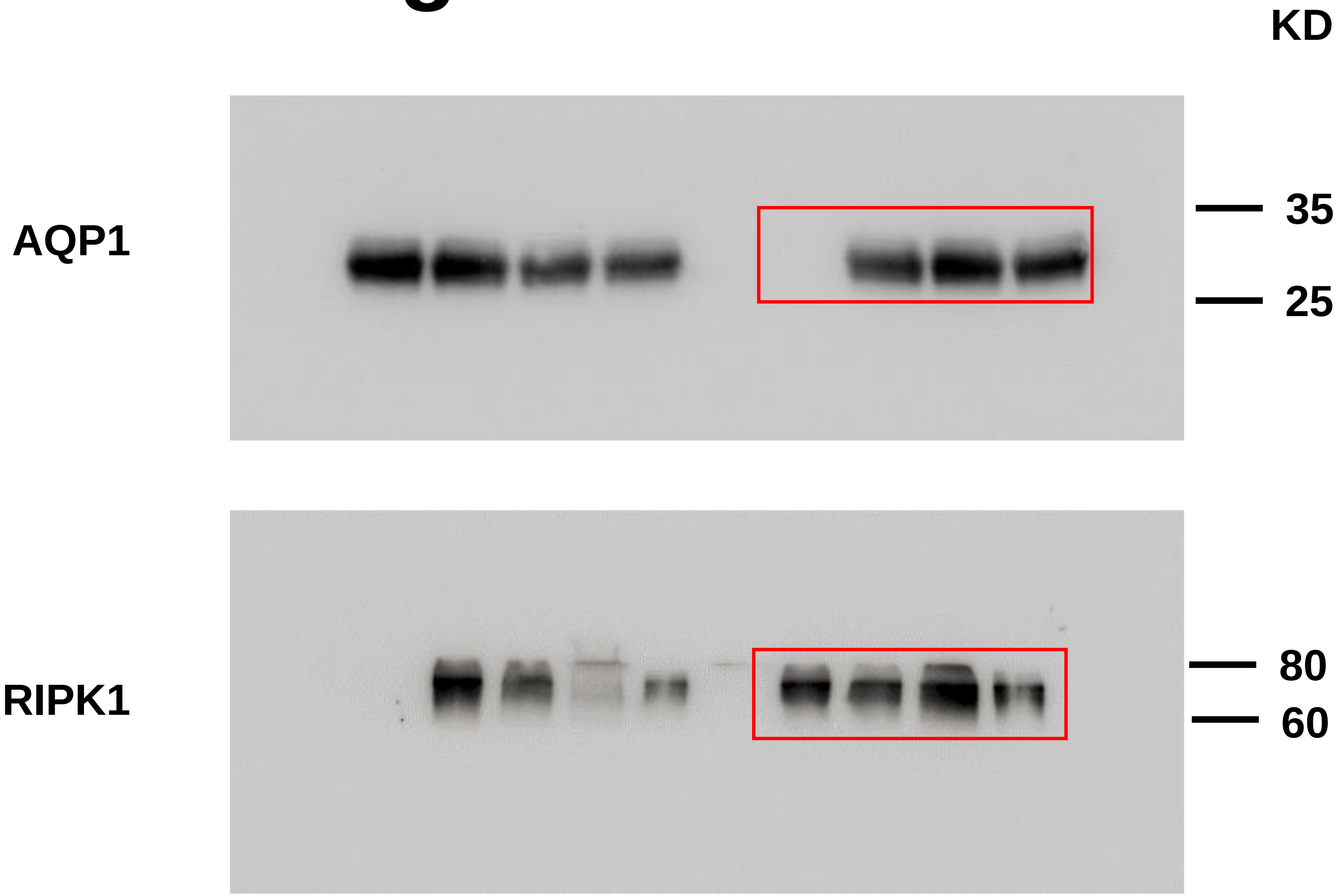

Figure 4A- 4T1

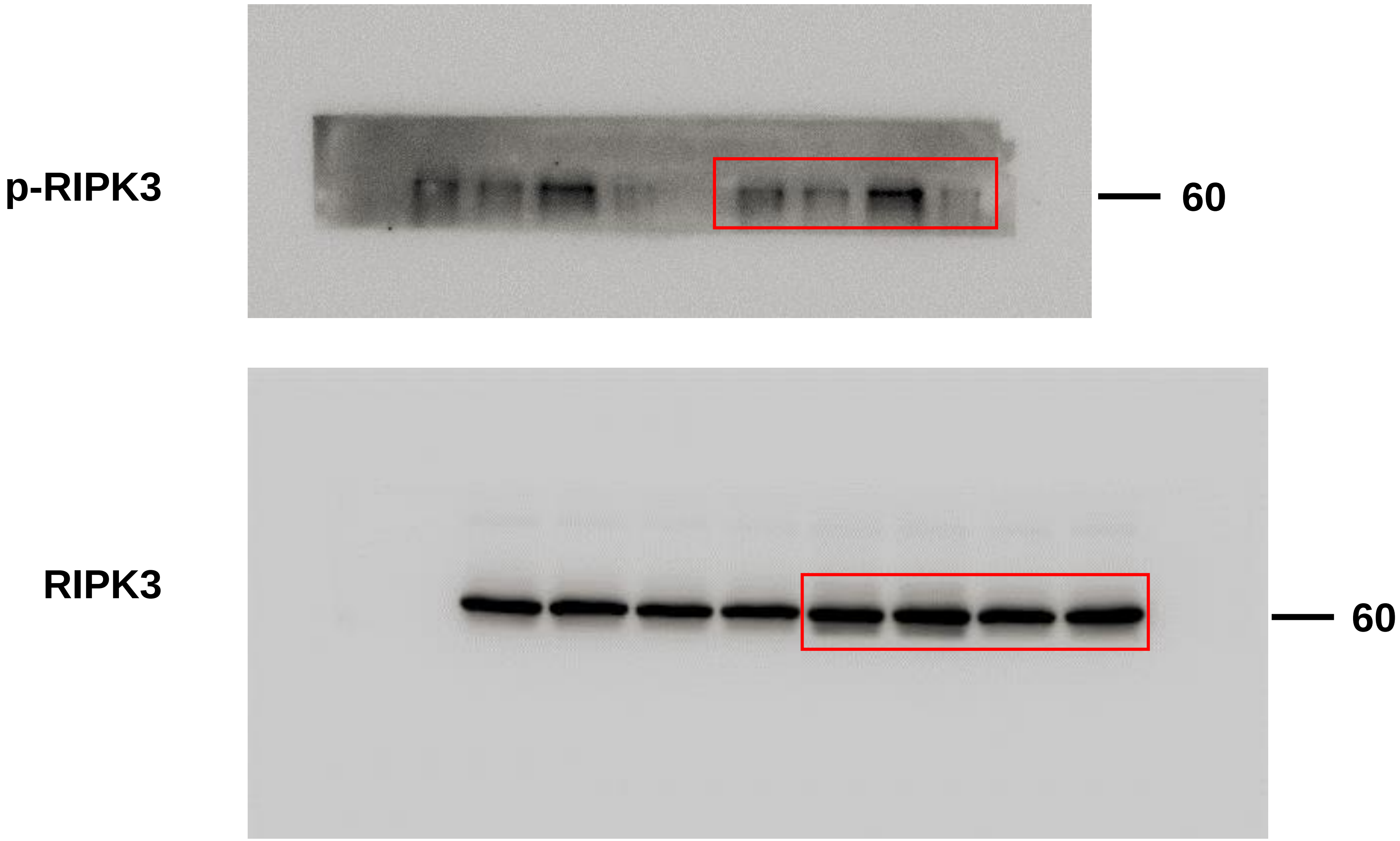

Figure 4A- 4T1

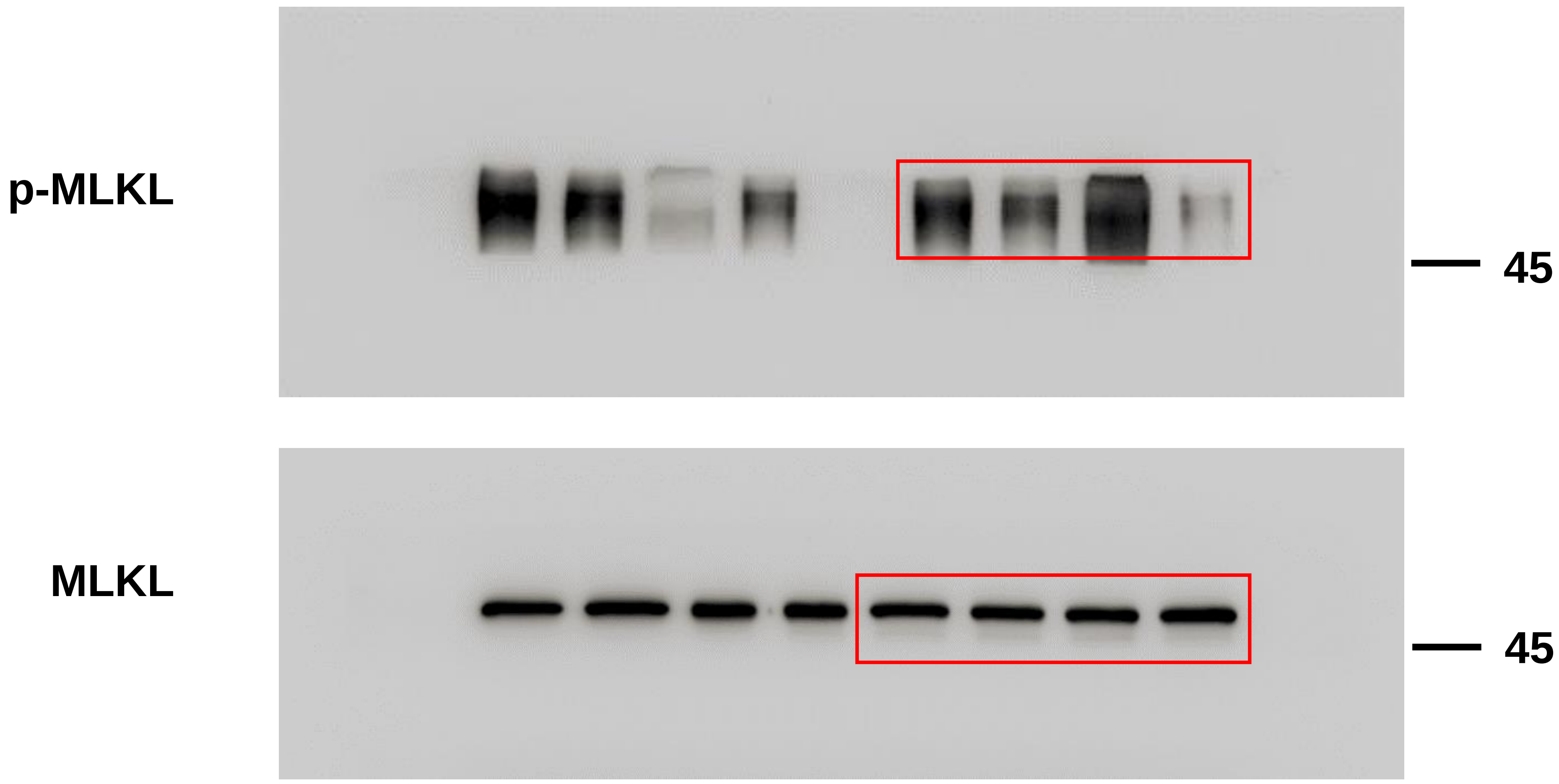

Figure 4A- 4T1

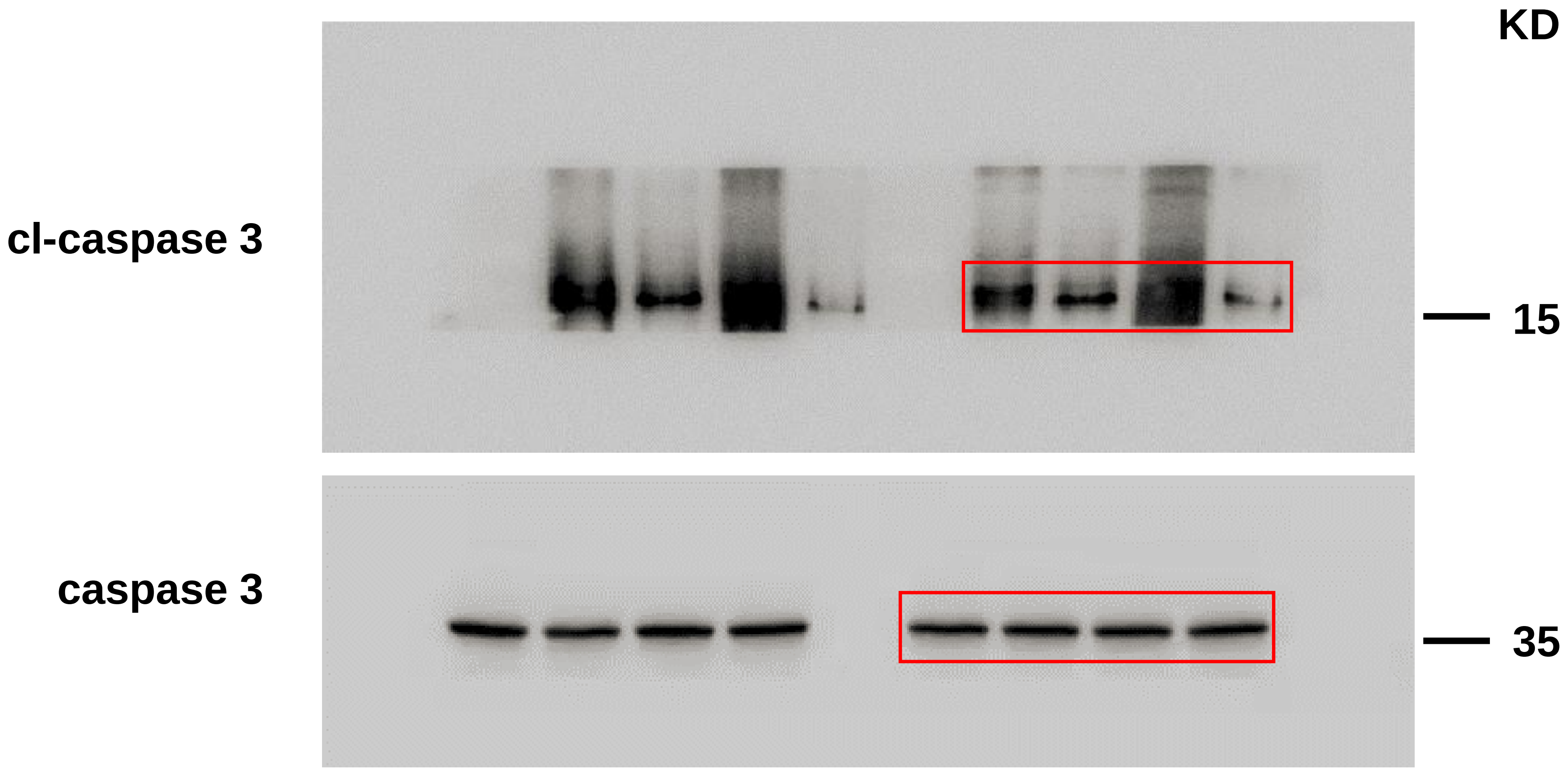

Figure 4A

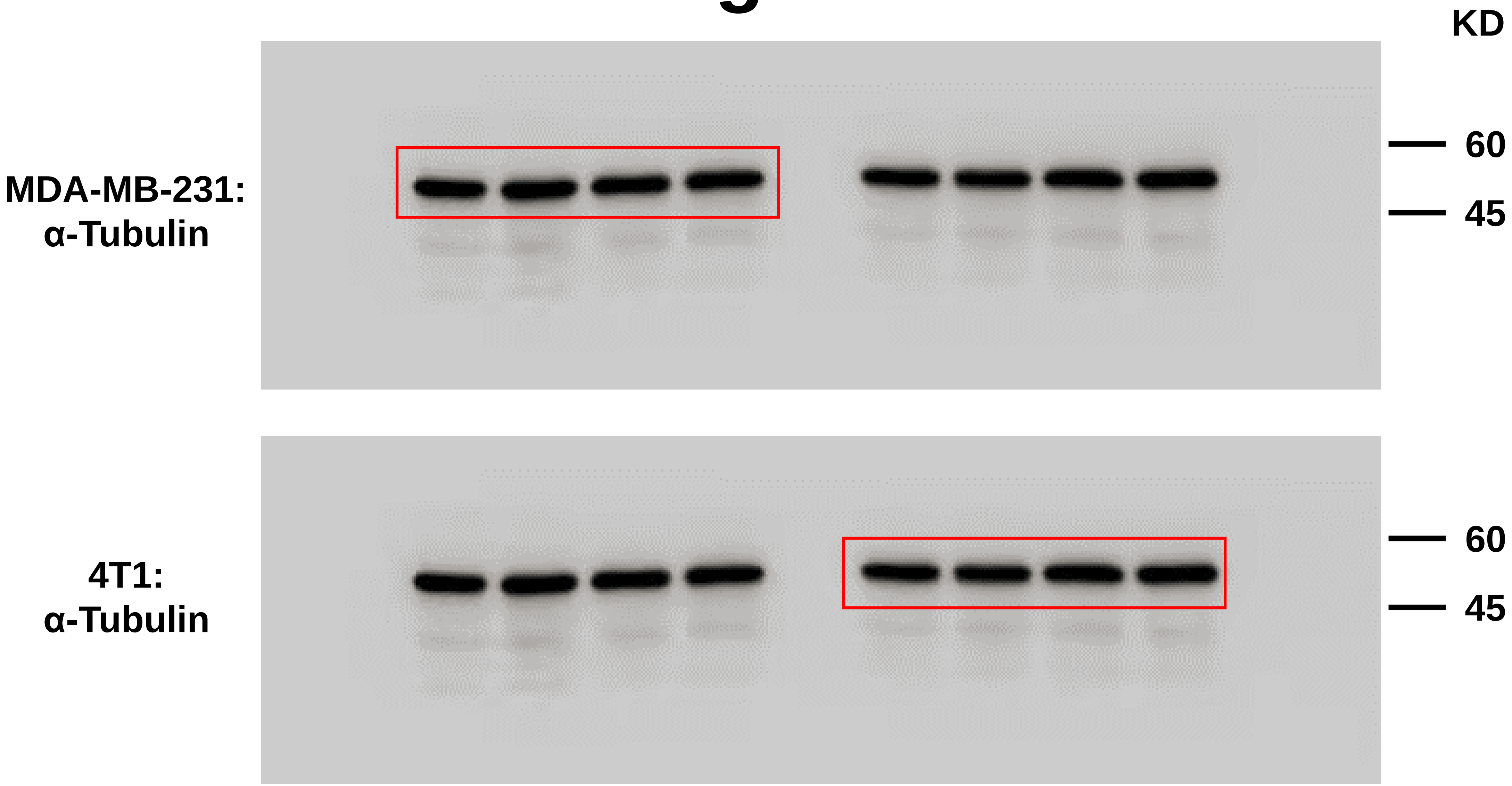

Figure 6B

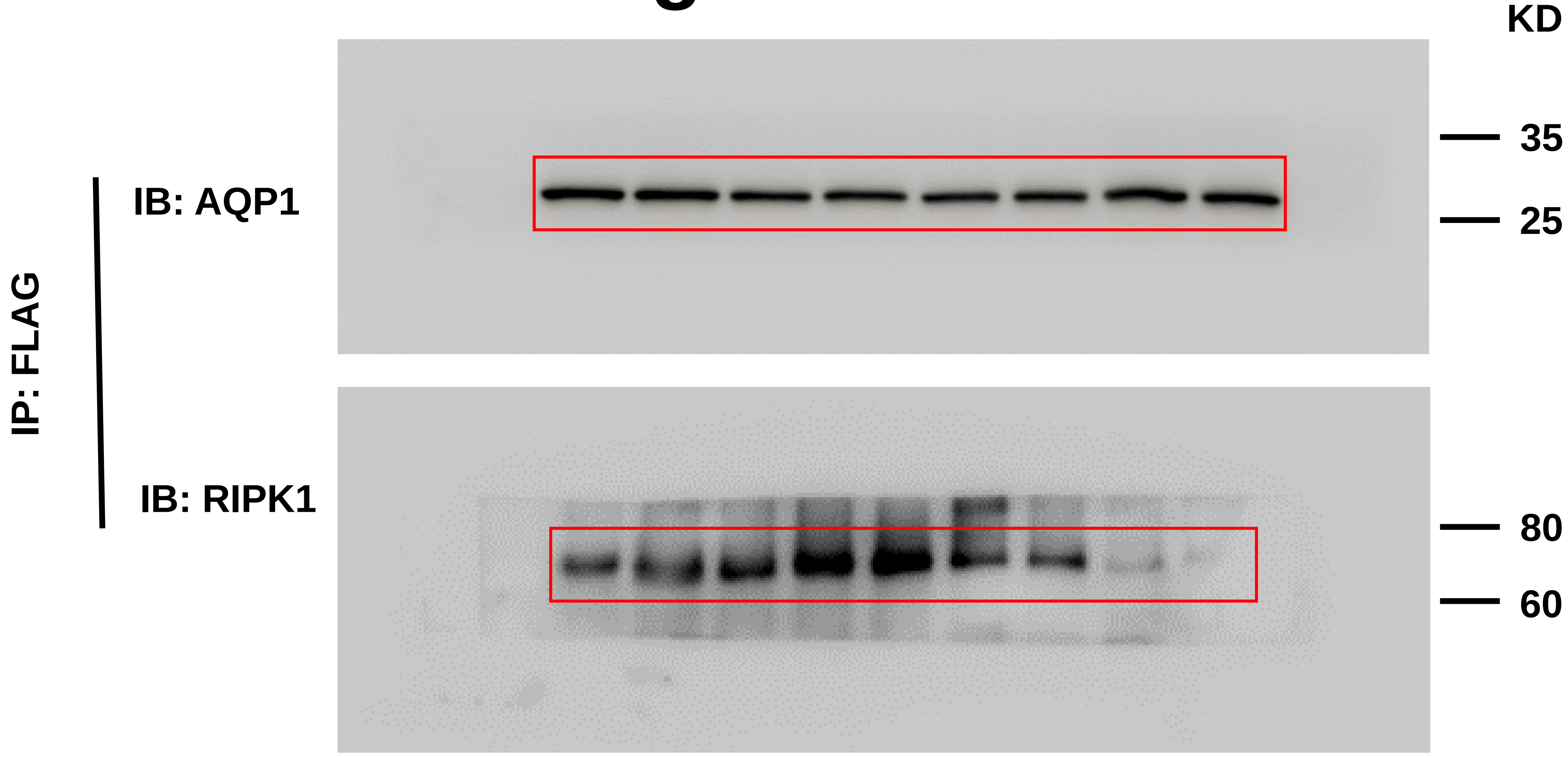

Figure 6B

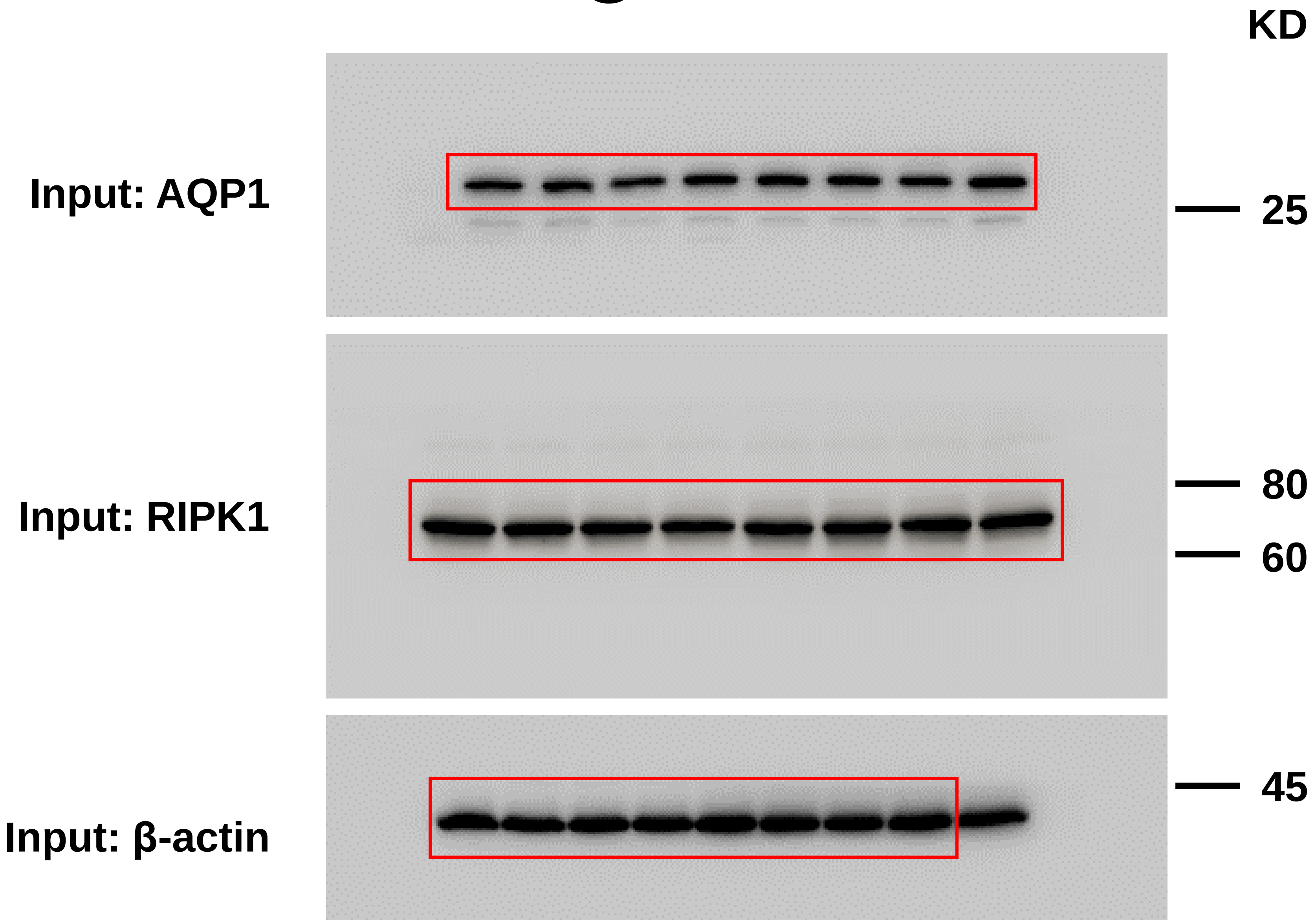

Figure 6C

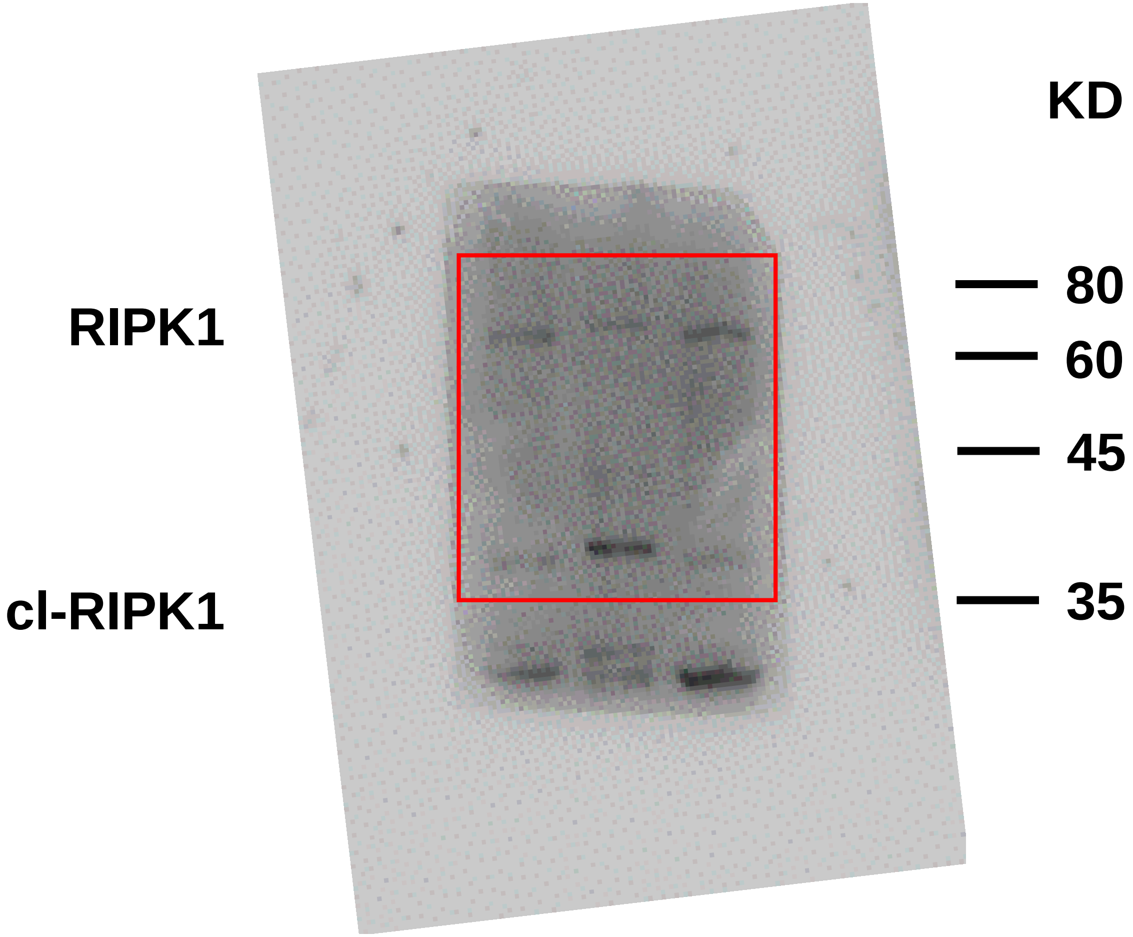

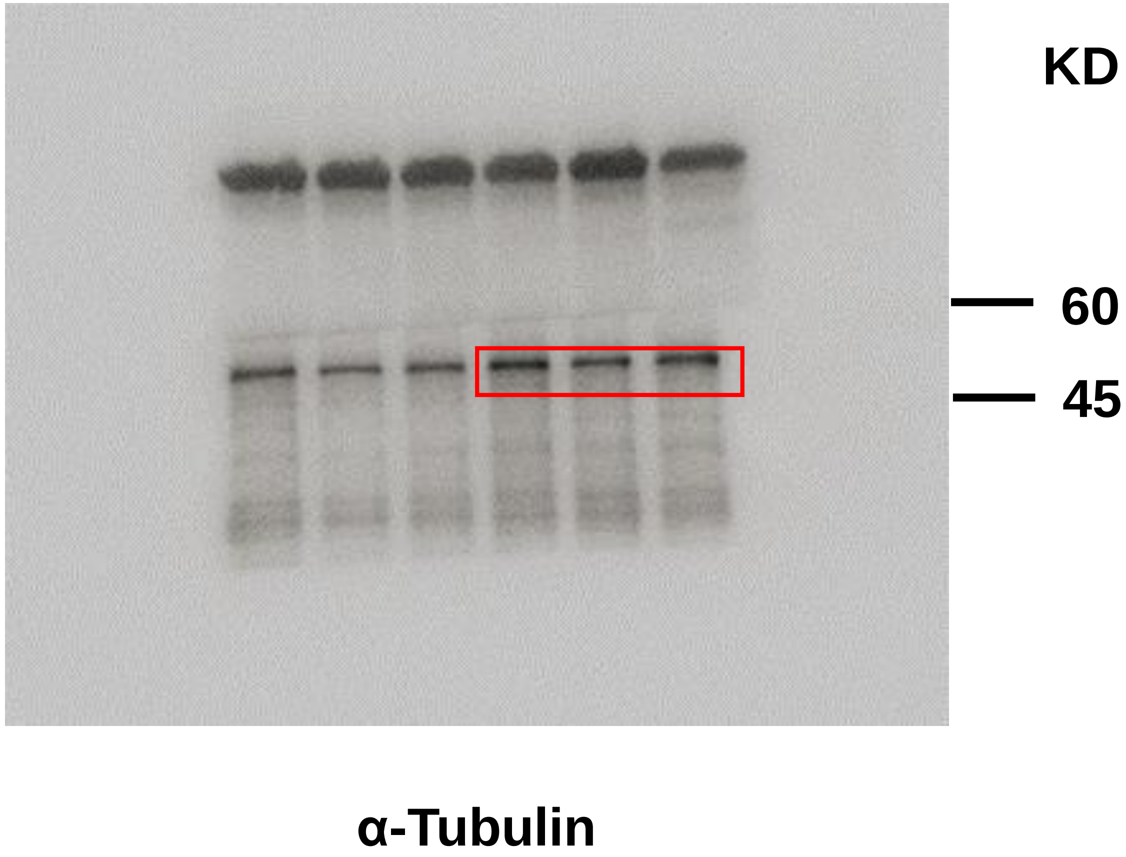

Supplementary Figure 3

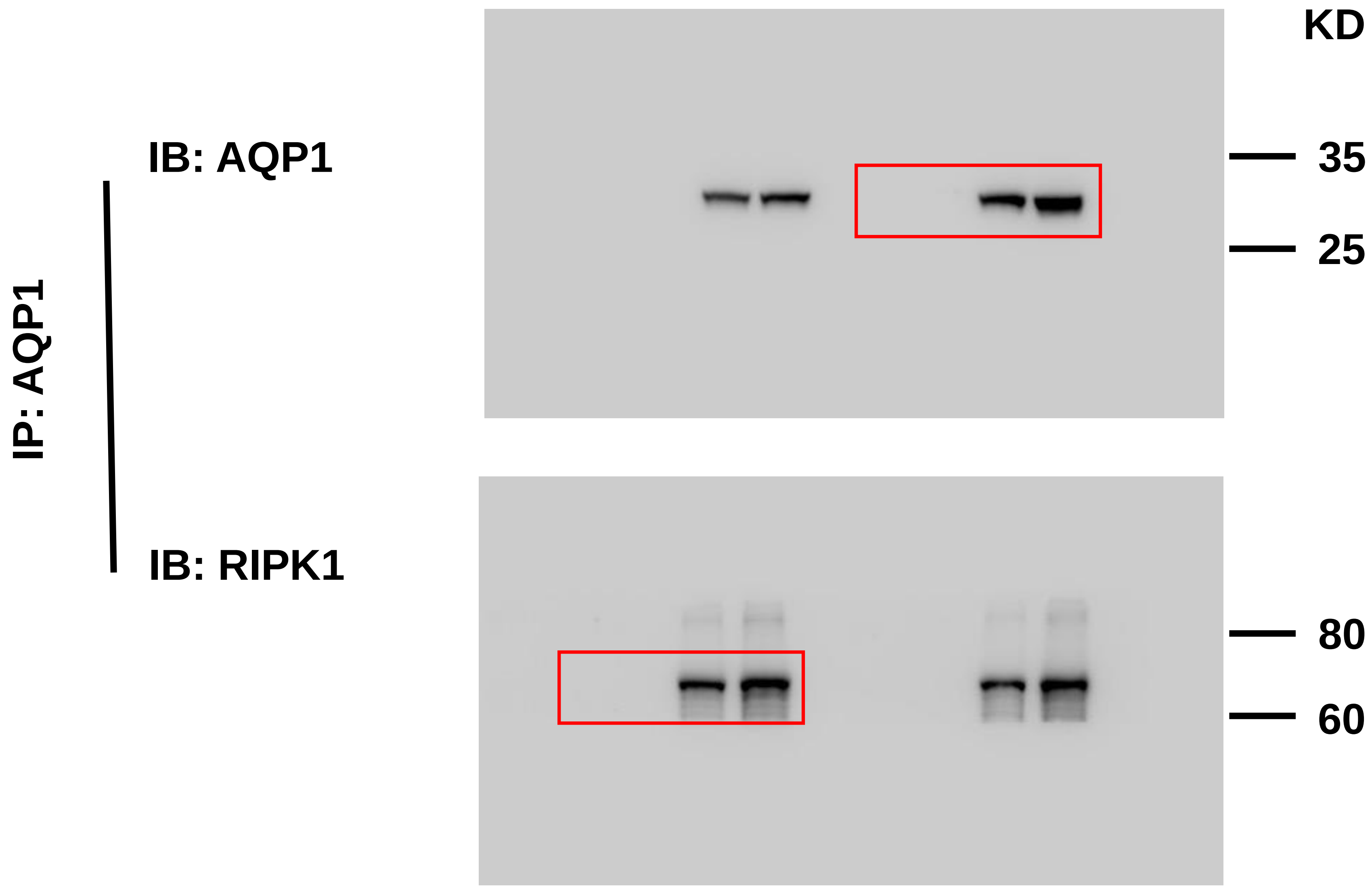

Supplementary Figure 3

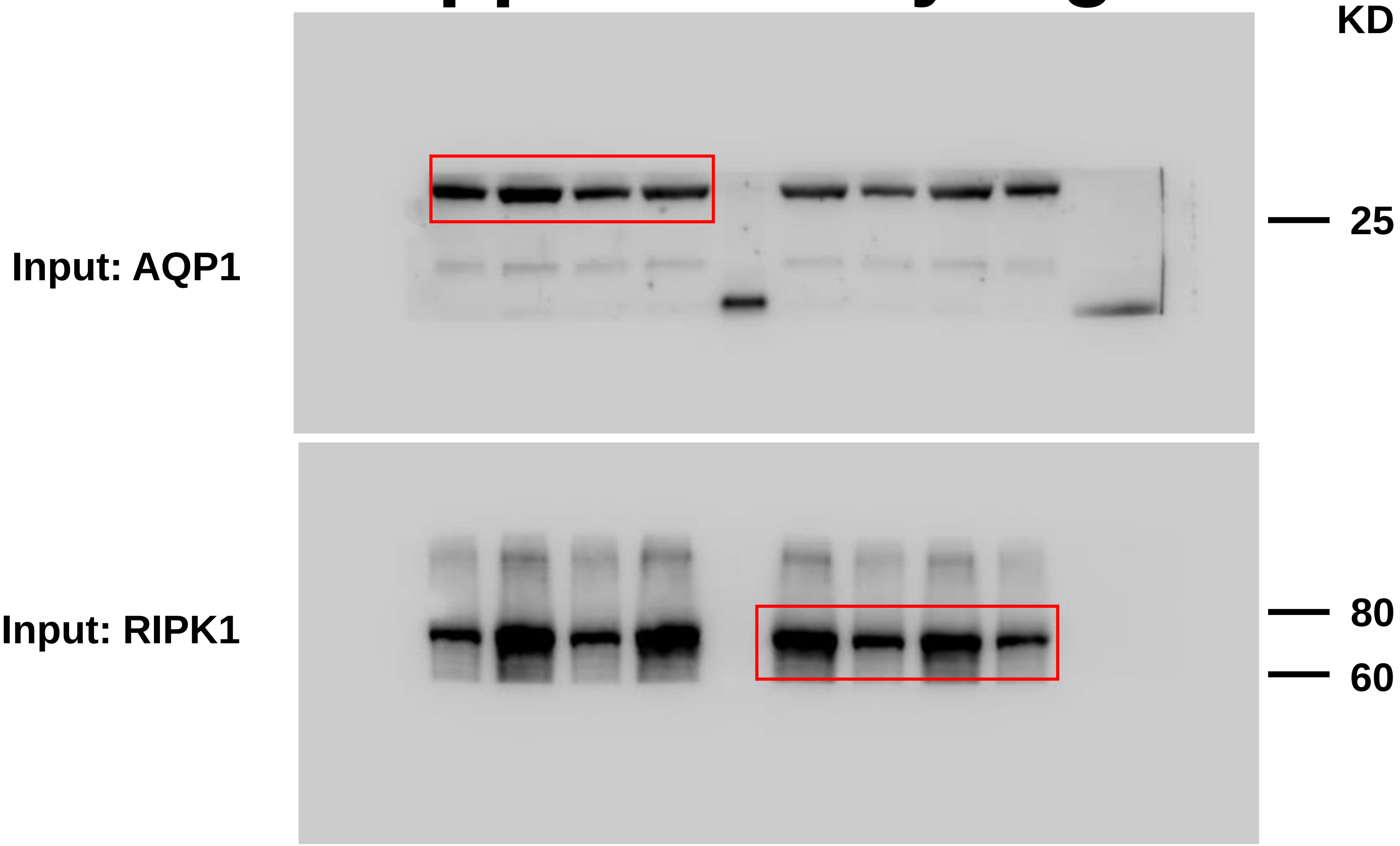

Supplementary Figure 3

Input: β-actin

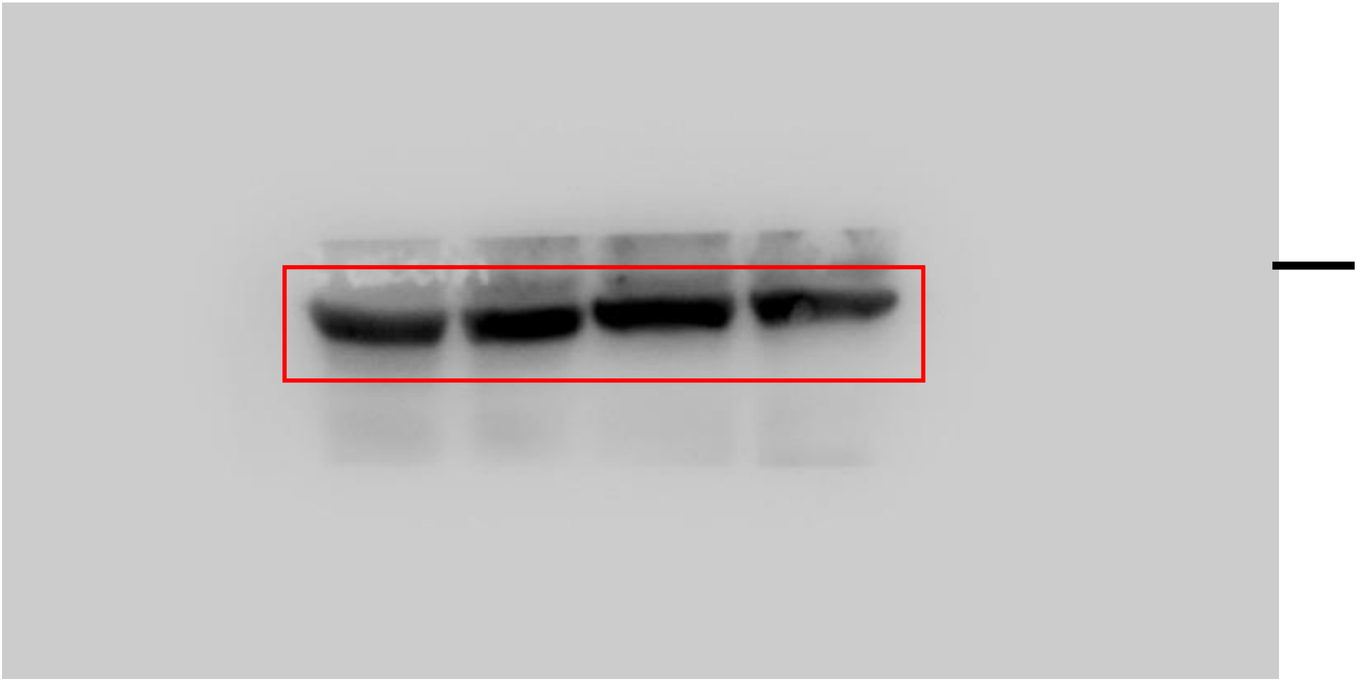

Supplemental Figure 5

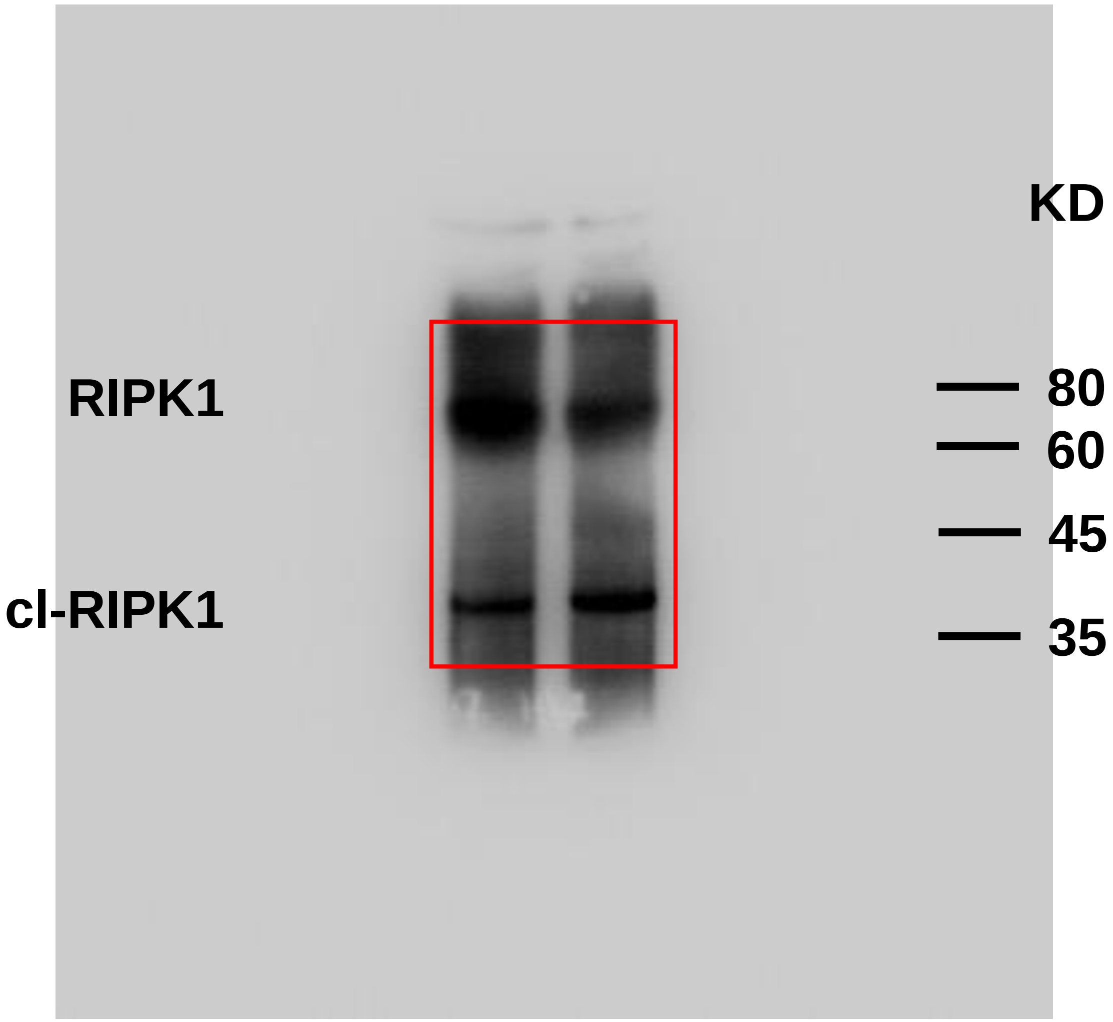

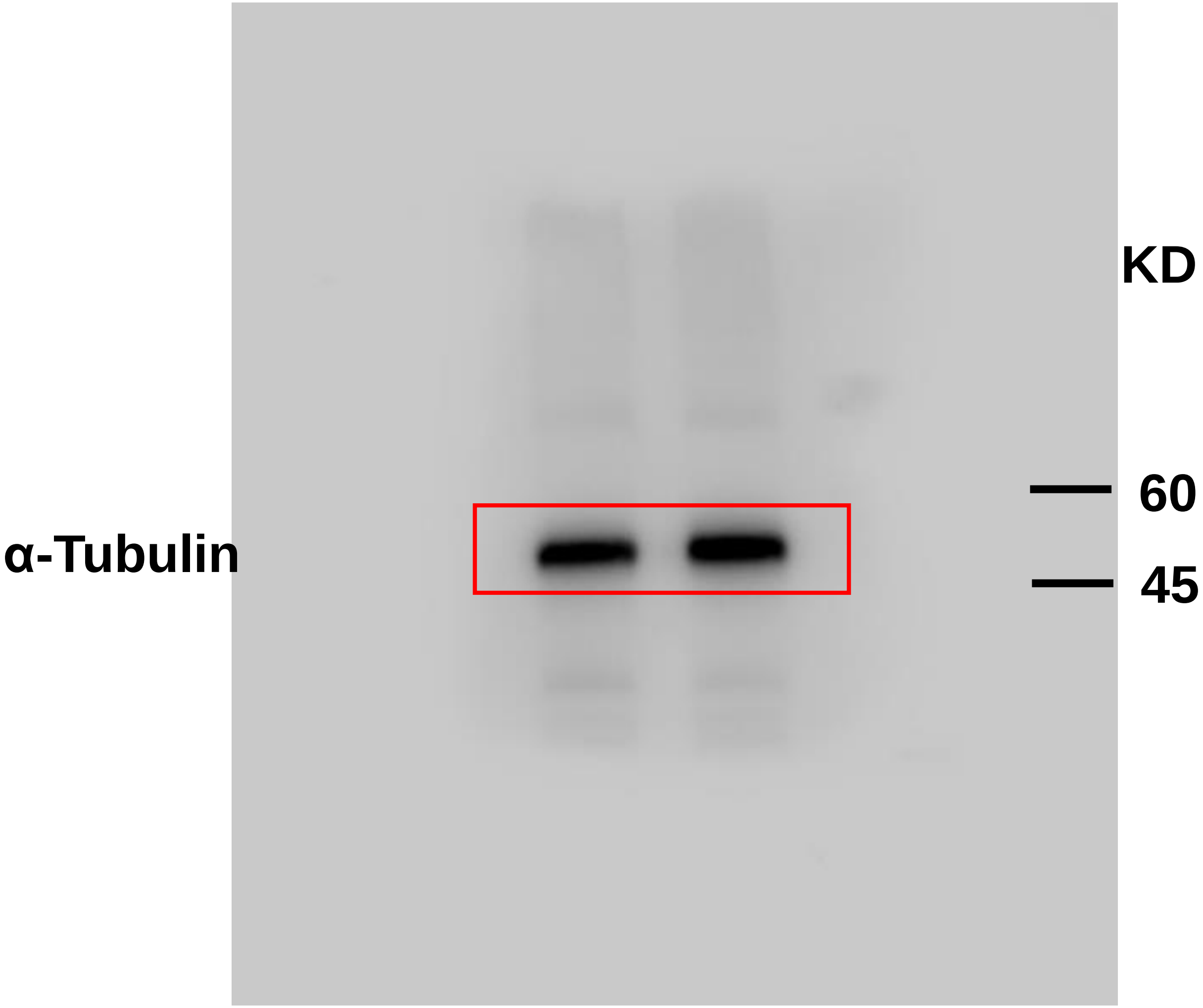

Supplement: Supplementary file 1 — Supplementary Information [file 41523_2021_261_MOESM1_ESM.pdf]
