# Supplementary material for: RIPK1 is a negative mediator in Aquaporin 1-driven triple-negative breast carcinoma progression and metastasis
Source: NPJ Breast Cancer. 2021 May 12;7:53. doi: 10.1038/s41523-021-00261-5 (PMC8115349; doi:10.1038/s41523-021-00261-5)
Supplement: Supplementary file 2 — Reporting Summary [file 41523_2021_261_MOESM2_ESM.pdf]

nature research

Corresponding author(s): Huiwen Ren; Fanxin Zeng

Reporting Summary

Nature Research wishes to improve the reproducibility of the work that we publish. This form provides structure for consistency and transparency in reporting. For further information on Nature Research policies, see Authors & Referees and the Editorial Policy Checklist.

Statistical parameters

When statistical analyses are reported, confirm that the following items are present in the relevant location (e.g. figure legend, table legend, main text, or Methods section).

n/a	Confirmed	
☐	☒	The exact sample size (*n*) for each experimental group/condition, given as a discrete number and unit of measurement
☐	☒	An indication of whether measurements were taken from distinct samples or whether the same sample was measured repeatedly
☐	☒	The statistical test(s) used AND whether they are one- or two-sided<br>*Only common tests should be described solely by name; describe more complex techniques in the Methods section.*
☐	☒	A description of all covariates tested
☐	☒	A description of any assumptions or corrections, such as tests of normality and adjustment for multiple comparisons
☐	☒	A full description of the statistics including central tendency (e.g. means) or other basic estimates (e.g. regression coefficient) AND variation (e.g. standard deviation) or associated estimates of uncertainty (e.g. confidence intervals)
☐	☒	For null hypothesis testing, the test statistic (e.g. *F*, *t*, *r*) with confidence intervals, effect sizes, degrees of freedom and *P* value noted<br>*Give P values as exact values whenever suitable.*
☒	☐	For Bayesian analysis, information on the choice of priors and Markov chain Monte Carlo settings
☒	☐	For hierarchical and complex designs, identification of the appropriate level for tests and full reporting of outcomes
☐	☒	Estimates of effect sizes (e.g. Cohen's *d*, Pearson's *r*), indicating how they were calculated
☐	☒	Clearly defined error bars<br>*State explicitly what error bars represent (e.g. SD, SE, CI)*

Our web collection on statistics for biologists may be useful.

Software and code

Policy information about availability of computer code

Data collection	Windows Office Excel 2013
Data analysis	GraphPad Prism version 8.01 and SPSS version 20.0

For manuscripts utilizing custom algorithms or software that are central to the research but not yet described in published literature, software must be made available to editors/reviewers upon request. We strongly encourage code deposition in a community repository (e.g. GitHub). See the Nature Research guidelines for submitting code & software for further information.

Data

Policy information about availability of data

All manuscripts must include a data availability statement. This statement should provide the following information, where applicable:

- Accession codes, unique identifiers, or web links for publicly available datasets
- A list of figures that have associated raw data
- A description of any restrictions on data availability

The public data resources included in the Oncomine database (http://www.oncomine.org), the Cancer Genome Atlas (TCGA, https://identifiers.org/cbioportal:brca_tcga), Genotype-Tissue Expression (GTEx, https://gtexportal.org), and the Gene Expression Omnibus data repository (GEO, https://identifiers.org/geo:GSE1456, https://identifiers.org/geo:GSE6532, and https://identifiers.org/geo:GSE7390). Institutional datasets supporting the figures and tables in the current

article are not publicly available to protect the patient privacy, but can be accessed from the corresponding author upon reasonable request. All other data supporting the findings of this study are available within the paper and supplemental materials. All the uncropped western blots generated during this study are available in Supplementary Figure 6.

Field-specific reporting

Please select the best fit for your research. If you are not sure, read the appropriate sections before making your selection.

☒ Life sciences ☐ Behavioural & social sciences ☐ Ecological, evolutionary & environmental sciences

For a reference copy of the document with all sections, see nature.com/authors/policies/ReportingSummary-flat.pdf

Life sciences study design

All studies must disclose on these points even when the disclosure is negative.

Sample size	62
Data exclusions	N/A
Replication	All the tests were replicated at least twice.
Randomization	N/A
Blinding	N/A

Reporting for specific materials, systems and methods

Materials & experimental systems

n/a	Involved in the study
☒	☐ Unique biological materials
☐	☒ Antibodies
☐	☒ Eukaryotic cell lines
☒	☐ Palaeontology
☐	☒ Animals and other organisms
☐	☒ Human research participants

Methods

n/a	Involved in the study
☒	☐ ChIP-seq
☒	☐ Flow cytometry
☒	☐ MRI-based neuroimaging

Antibodies

Antibodies used	Mouse monoclonal antibodies against AQP1 (ab9566) and RIPK1 (ab72139) were purchased from Abcam (Cambridge, MA). Mouse monoclonal antibodies against RIPK1 (610458), Caspase-8 (9746), and RIPK3 (sc-374639) were bought from BD Biosciences (San Jose, CA), Cell Signaling Technology (Danvers, MA), and Santa Cruz Biotechnology (Dallas, TX), respectively. The rabbit monoclonal antibody against p-MLKL (S345) (ab196436), p-RIPK3 (S227) (ab209384) and p-RIPK3 (S232) (ab195117) and rabbit polyclonal antibody against RIPK1 (ab106393) and MLKL (ab194699) were purchased from Abcam. The rabbit polyclonal antibody against cleaved caspase-3 (9661) and caspase-3 (9662) were bought from Cell Signaling Technology. The rabbit monoclonal anti-β-actin antibody (AC026) and anti-α-tubulin antibody (AC013) were from Abclonal (Wuhan, China).
Validation	The validation of antibodies was conformed to the manufacturers' instruction.

Eukaryotic cell lines

Policy information about cell lines

Cell line source(s)	The MDA-MB-231 cells (No. 3111C0001CCC000014) were obtained from the Cell Resource Center of Institute of Basic Medicine, Chinese Academy of Medical Sciences (Beijing, China). The HEK-293T cells (No. 3131C0001000200017) and 4T1 cells (No. 3131C0001000800032) were purchased from the Cell Resource Center, Shanghai Institutes for Biological Sciences, Chinese Academy of Sciences (Shanghai, China).
Authentication	None of the cell lines used in this study were authenticated.

Mycoplasma contamination	All cell lines tested negative for mycoplasma contamination.
Commonly misidentified lines (See ICLAC register)	N/A

Animals and other organisms

Policy information about studies involving animals; ARRIVE guidelines recommended for reporting animal research

Laboratory animals	seven-week-old female BALB/c mice
Wild animals	N/A
Field-collected samples	The mice were maintained on a commercial pellet diet, given deionized water ad libitum, and kept in plastic cages in a 20±2℃ room at 50–70% relative humidity with a 12-hour light/dark cycle. All the mice were sacrificed by cervical dislocation at the end of experiment unless they died of cancer metastasis.

Human research participants

Policy information about studies involving human research participants

Population characteristics	All the patients involved were female and diagnosed of triple-negative breast cancer. The average age was 45.4 yo. Please see details in Table 1.
Recruitment	The cases were consecutively recruited between May 1, 2012 and April 30, 2013 at Tianjin Medical University Cancer Institute and Hospital and the First Affiliated Hospital of Xiamen University. No selection bias existed in the present study.
